# Effects of traditional Chinese medicine non-pharmacological intervention in patients with simple obesity: a systematic review and network meta-analysis

**DOI:** 10.3389/fmed.2026.1815757

**Published:** 2026-05-08

**Authors:** Yuqi Wang, Lina Wang, Fang Cheng, Xiaohong Yuan, Minfang Zhu, Min Xu, Nafei Tu

**Affiliations:** 1 Zhejiang Chinese Medical University, Hangzhou, Zhejiang, China; 2 Department of Hematology, The First Affiliated Hospital of Zhejiang Chinese Medical University (Zhejiang Provincial Hospital of Chinese Medicine), Hangzhou, Zhejiang, China; 3 The First Affiliated Hospital of Zhejiang Chinese Medical University (Zhejiang Provincial Hospital of Chinese Medicine), Hangzhou, Zhejiang, China

**Keywords:** BMI, network meta-analysis, non-pharmacological interventions, simple obesity, traditional Chinese medicine appropriate techniques

## Abstract

**Background:**

Obesity is a complex chronic disease involving genetic, environmental, and behavioral factors. Traditional Chinese medicine (TCM) non-pharmacological therapies are promising complementary strategies for simple obesity, yet evidence is fragmented and their comparative effectiveness remains unclear.

**Objectives:**

This study aimed to evaluate and compare the efficacy of different TCM non-pharmacological interventions for simple obesity.

**Methods:**

A comprehensive literature search was performed in PubMed, EMBASE, Web of Science, the Cochrane Central Register of Controlled Trials (CENTRAL), China National Knowledge Infrastructure (CNKI), Wanfang Data, VIP, and the China Biomedical Literature Database (CBM) on December 31, 2025. Effect sizes were estimated using standardized mean differences (SMD) with random-effects models, methodological quality was assessed via the Cochrane Handbook version 5.1.0, and network meta-analysis (NMA) was conducted under a frequentist framework using Stata 18.0.

**Results:**

A total of 37 randomized controlled trials (RCTs) involving 2,832 patients were included, examining six TCM non-pharmacological interventions and two control conditions. NMA demonstrated that all six interventions improved body mass index (BMI) and waist circumference (WC) to varying degrees versus usual care and placebo. Compared to placebo, moxibustion was associated with significantly greater reductions in BMI (SMD = −1.75, 95%CI: −2.68 to −0.83, surface under the cumulative ranking curve [SUCRA] = 98.8%). Relative to control, moxibustion was associated with significantly greater reductions in WC (SMD = −1.48, 95%CI: −2.31 to −0.64, SUCRA = 90.0%).

**Conclusion:**

Current evidence indicates that different non-pharmacological TCM interventions may have differential effects on body mass index and waist circumference in adults with simple obesity, with moxibustion showing a possible relative advantage. Owing to low evidence quality and study limitations, findings should be interpreted cautiously.

**Systematic review registration:**

https://www.crd.york.ac.uk/PROSPERO/, PROSPERO ID CRD42024614904.

## 1 Introduction

Obesity has become a major public health challenge worldwide. The World Health Organization (WHO) has identified it as a priority area for intervention because it not only markedly increases the risk of cardiovascular and cerebrovascular diseases, diabetes, and malignancies, but also leads to a range of mental health problems and imposes a substantial social and economic burden (1, 2). Among the various types of obesity, simple obesity accounts for approximately 95% of all cases; it is characterized by abnormal body fat accumulation in the absence of neuroendocrine lesions and is considered non-pathological obesity (3). Epidemiological projections indicate that, by 2030, the number of people with obesity worldwide will exceed 2 billion, with the number in China alone reaching 800 million, and obesity-related medical expenditures are expected to account for 22% of total national health spending (4–6). In response to this alarming situation, WHO launched the Acceleration Plan to STOP Obesity in 2022, emphasizing the creation of a multidimensional prevention and control system (7). In 2024, China, in collaboration with multiple sectors, initiated the “National Healthy Weight Management Year”, further underscoring the urgency of obesity prevention and control.

It is of great significance to explore effective strategies for the treatment and management of obesity. Current interventions for obesity mainly include pharmacotherapy, bariatric surgery, and non-pharmacological interventions (8–10). Although pharmacotherapy is widely used, it is often associated with adverse reactions such as gastrointestinal discomfort and cardiovascular risk, and the weight regain rate after drug cessation is high (11). Bariatric surgery achieves significant weight loss but has limitations including surgical trauma, postoperative nutritional complications, and irreversibility. Therefore, it is only suitable for individuals with severe obesity and is difficult to promote on a large scale (12). As a core component of non-pharmacological interventions, lifestyle interventions centered on dietary modification, exercise guidance, and behavioral modification are recognized as the cornerstone of obesity management. However, existing studies and clinical guidelines indicate that lifestyle intervention alone also struggles to achieve long-term stable weight loss. On the one hand, patient compliance is generally insufficient, with only 11% of overweight or obese individuals adhering to long-term diet and exercise regimens (13). On the other hand, even if short-term weight loss is attained, weight regain remains a prominent issue, and weight maintenance rates are far from satisfactory. The inherent limitations of pharmacotherapy, surgery, and lifestyle interventions have contributed to the current dilemma in obesity management (14). Therefore, there is an urgent need to explore complementary interventions that are effective, safe, and well-tolerated, so as to establish a more personalized and multi-dimensional comprehensive model for obesity management.

As an important complementary and alternative therapy, traditional Chinese medicine (TCM) non-pharmacological intervention has received increasing attention in the intervention of obesity. Among them, acupressure, moxibustion, acupoint catgut embedding and auricular acupuncture have been widely used in East Asia (15–17). Although a series of randomized controlled trials (RCTs) have been conducted to evaluate the effects of various non-pharmacological interventions on obesity and related metabolic disorders, the findings remain inconsistent, limiting their clinical application. In addition, several systematic reviews and meta-analyses have preliminarily explored the effectiveness of single non-pharmacological interventions in improving body weight, body mass index and waist circumference in individuals with obesity (18, 19). However, conventional pairwise meta-analyses are limited to comparing only two interventions at a time, making it difficult to comprehensively evaluate and rank the relative efficacy of multiple non-pharmacological strategies. Furthermore, some existing network meta-analyses have only focused on the impacts of one single type of TCM non-pharmacological interventions on obesity, including different traditional Chinese exercises (20) or various acupuncture interventions, and failed to assess other types of TCM non-pharmacological interventions simultaneously. In contrast, network meta-analysis (NMA) can further rank the efficacy of different interventions, thereby helping identify the optimal non-pharmacological strategies for the clinical management of obesity.

Therefore, this study aims to systematically synthesize evidence from RCTs and perform a NMA to compare and rank the effectiveness of various TCM non-pharmacological interventions for obesity management, with body mass index (BMI) and waist circumference (WC) as core outcomes. The findings aim to provide evidence-based guidance for healthcare professionals and facilitate the clinical application of optimal individualized TCM non-pharmacological interventions.

## 2 Methods

### 2.1 Registration

This protocol has been developed in accordance with the Preferred Reporting Items for Systematic Reviews and Meta-Analyses Protocols (PRISMA-P) guidelines to ensure methodological transparency and rigour. The protocol is registered in the International Prospective Register of Systematic Reviews PROSPERO (CRD42024614904).

### 2.2 Search strategy

We searched PubMed, EMBASE, Web of Science, and the Cochrane Central Register of Controlled Trials (CENTRAL); China National Knowledge Infrastructure (CNKI), Wanfang Data, VIP Information Chinese Journal Service Platform, and the China Biomedical Literature Database (CBM) from the dates of their respective inceptions until December 31, 2025. Two reviewers developed the basic search strategy as follows: (obesity* OR simple obesity* OR overweight* OR adipose* OR body mass index* OR weight gain*) AND (acupuncture* OR acupoint catgut embedding * OR moxibustion* OR massage* OR traditional exercise* OR auricular acupuncture) AND random*. The search strategy is shown comprehensively in Supplementary Appendix 2. We also traced the references of included RCTs and relevant reviews to identify any potentially eligible studies. There were no restrictions in terms of publication status and publication date.

### 2.3 Eligibility criteria

*Population*: Participants were adults aged 18 to 65 years who were diagnosed with simple obesity according to the Chinese Guidelines for the Prevention and Treatment of Overweight and Obesity in Adults and had a body mass index (BMI) of 24 kg/m^2^ or higher (21). To rule out confounding factors, participants were required to be free of any established coexisting conditions (e.g., hypertension, diabetes) and to have no clinical symptoms or signs of a related noncommunicable chronic disease.

*Study design*: RCTs.

*Interventions*: We included all TCM non-pharmacological interventions and categorized them into relevant groups. On the basis of routine diet and exercise interventions, the experimental group received a single TCM non-pharmacological therapy, including massage, auricular acupuncture, acupuncture, moxibustion, acupoint catgut embedding, and traditional Chinese exercises.

*Comparators*: Eligible studies included at least one experimental group and one passive control group receiving standard care. The control intervention could be either an inactive (placebo) or an active intervention, provided that the experimental group received the same active components concurrently.

*Outcomes*: At least one of BMI and WC were included.

### 2.4 Study selection

EndNote X7 software was used to manage literature search records. According to the eligibility criteria, two reviewers (Y. Q. W. and N. F. T.) independently screened the titles and abstracts of all identified records after duplicate removal. All potentially eligible studies and any overlapping studies underwent full-text evaluation. Any disagreements between the two reviewers were resolved through discussion. If discrepancies persisted, a third reviewer (M. X.) made the final decision.

### 2.5 Data extraction

Paired evaluators independently used standardized data-extraction tables to extract data on the following baseline characteristics and outcomes of interest: name of the first author, year of publication, country, diagnostic criteria for simple obesity, outcome measure, sample, mean age, mean baseline, and details of the intervention.

### 2.6 Quality and certainty of evidence assessment

Two authors (L. N. W. and F. C.) assessed the risk of bias of the included studies in accordance with the Cochrane Handbook for Systematic Reviews of Interventions (version 5.1.0) (22). The domains assessed included random sequence generation, allocation concealment, blinding of participants and personnel, blinding of outcome assessment, incomplete outcome data, selective reporting, and other potential sources of bias. Each domain was judged as low, unclear, or high risk of bias. A study was classified as having an overall high risk of bias if it was rated as high risk in any domain; otherwise, it was classified as low risk of bias. Any disagreements between reviewers were resolved through discussion.

The overall risk of bias for each study was graded as “low,” “some concerns,” or “high.” The certainty of evidence for BMI and waist circumference reduction was evaluated using the Confidence in Network Meta-Analysis (CINeMA) framework, which includes six domains: within-study bias, reporting bias, indirectness, imprecision, heterogeneity, and incoherence (23, 24). For each pairwise comparison, the overall certainty was rated as “very low,” “low,” “moderate,” or “high.” Two independent reviewers (N. F. T. and M. F. Z.) performed assessments and ratings; any disagreements were resolved via discussion with a third reviewer (M. X.) until consensus was reached.

### 2.7 Data analysis

The initial analysis involved extracting all comparative data from the included studies. All outcomes were converted to standard units, and the mean difference was calculated by subtracting the pre-training mean from the post-training mean. Continuous variables were reported as means (SDs). Pooled effect estimates were reported with corresponding 95% confidence intervals (CI) and 95% prediction intervals. When only 95% confidence intervals (CIs) or standard errors (SEs) were reported, or when changes in means and standard deviations (SDs) before and after the intervention were not provided, SEs and CIs were converted and SDs were estimated in accordance with the guidelines outlined in the Cochrane Handbook (25).

Statistical analyses were performed using Stata 18.0 software. A random-effects multivariate network meta-analysis (NMA) was conducted within a frequentist framework. Considering potential heterogeneity across studies, all comparisons were analyzed using a random-effects model (26). Heterogeneity was quantified using the Cochran Q test and the Higgins I^2^ statistic, with I^2^ values of <25, 25–75%, and >75% indicating low, moderate, and high heterogeneity, respectively. Network geometry was visualized using a network plot, in which node size was proportional to the total sample size of each intervention and edge thickness reflected the number of studies contributing to each direct comparison. Global inconsistency was assessed using the design-by-treatment interaction model to examine whether systematic differences existed between direct and indirect evidence across the entire network. Local inconsistency was subsequently evaluated using the node-splitting method, which compares direct and indirect effect estimates for specific intervention pairs to identify potential inconsistency at the pairwise level (27). A *p*-value > 0.05 in either test was interpreted as indicating no significant inconsistency. The relative effectiveness of each non-pharmacological intervention for BMI and WC was ranked using the surface under the cumulative ranking curve (SUCRA), which ranges from 0 to 100%. A higher SUCRA value indicates a greater probability that an intervention is among the most effective. Potential publication bias was assessed using Egger’s test and visual inspection of a comparison-adjusted funnel plot (28). Nevertheless, the clinical relevance of this apparent advantage depends on how effect size metrics, whether standardized mean difference (SMD) or mean difference (MD), are interpreted, along with their corresponding 95% CIs. Pre-specified sensitivity analyses were conducted to confirm the robustness of the primary findings, and pre-specified subgroup analyses were performed to explore effect modification by intervention duration, treatment frequency, and sample size. In addition, post-hoc meta-regression analyses were carried out to explore potential sources of heterogeneity and to determine whether other trial-level characteristics (e.g., baseline BMI) could account for the observed variability in therapeutic efficacy.

## 3 Results

### 3.1 Literature selection

**Figure 1:**
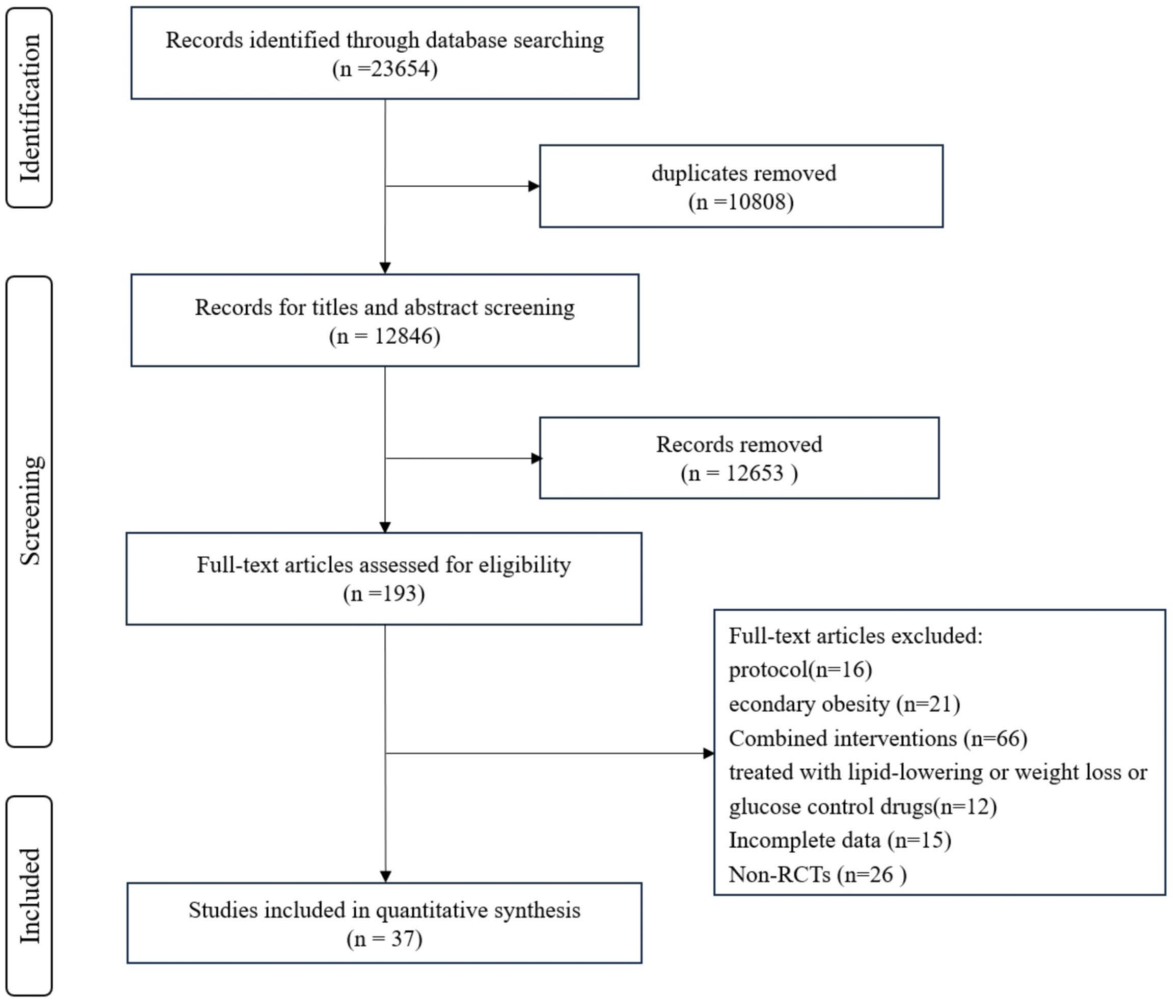
Flow diagram.

The initial literature search retrieved 23,654 records. Following the removal of 20,808 duplicates, 2,846 records underwent title and abstract screening. Of 193 full-texts screened, 156 were excluded and 37 were included (29–65). The detailed selection process is described in Figure 1.

### 3.2 Characteristics of the included studies

**Figure 2:**
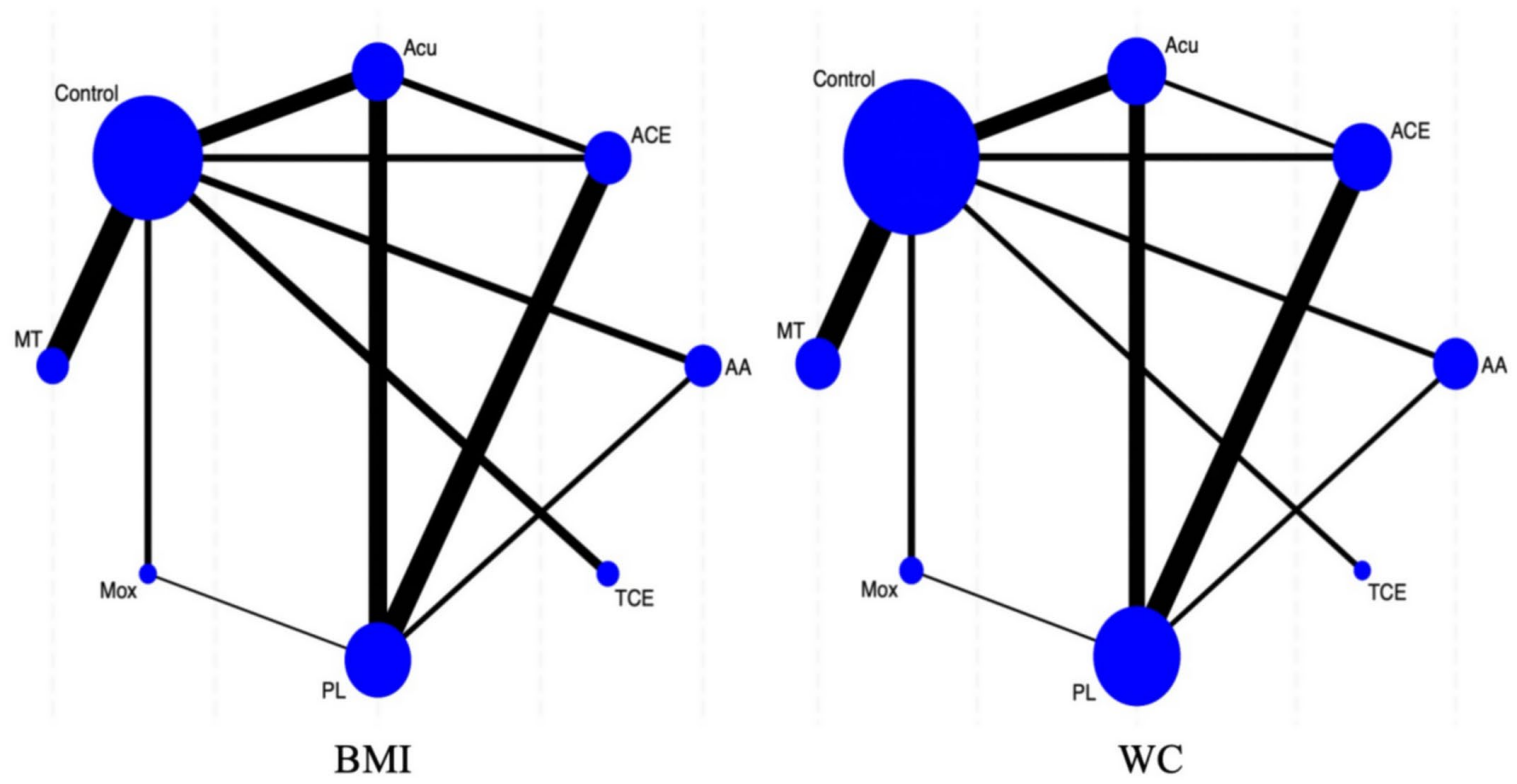
Network graph. BMI, body mass index; WC, waist circumference; PL, placebo therapy; Control, usual care; TCE, traditional Chinese exercises; Mox, moxibustion; MT, manual therapy; ACE, acupoint catgut embedding; AA, auricular acupuncture; Acu, acupuncture.

Table 1 presents the characteristics of the included studies. The studies were published between 2012 and 2025 and included 2,832 participants with a mean age ranging from 18 to 65 years. Most of the included studies were two-armed, and all 37 studies had an intervention duration of 4 weeks or longer. Geographically, most trials were conducted in Asia, predominantly in China (87.5%), with additional studies performed in Iran, Korea, Turkey, and Brazil. The interventions were distributed as follows: acupuncture (27.03%), auricular acupuncture (18.92%), acupoint catgut embedding (18.92%), moxibustion (8.11%), manual therapy (16.22%), and traditional Chinese exercises (10.80%). The most common intervention duration was 8 weeks (35.1%), and treatment was typically administered three times per week. Primary outcome measures consistently included body composition indices such as BMI and WC. Control groups mainly received usual care combined with lifestyle counseling or sham interventions. Thirty-seven studies reported BMI, and 32 reported WC. Figure 2 graphically displays the evidence networks for all treatment outcomes.

**Table 1:** Characteristics of included studies.

Study	Country	Sample E/C	Mean age (years)	Interventions	Treatment duration (weeks)	Intervention description	Outcome measures
Lien CY (33) 2012	China (Taiwan)	24/23	42.20 ± 12.40	Auricular acupuncture	4	All subjects were asked to receive three treatments a week for 4 weeks. Acupoints on only one ear were attended at each treatment followed by those on the other ear at the following visit. Hence, each ear underwent six treatments alternatively for the total of 12 treatments for each subject.	BMI, WC
			40.70 ± 9.70	Sham auricular acupuncture		Received sham auricular acupuncture using placebo plasters, The treatment procedure was the same as the Auricular acupuncture group.	
Güçel F (40) 2012	Turkey	20/20	34.60 ± 6.30	Acupuncture	5	Received two sessions of 20 min/week for a total of 10 sessions.	BMI
			36.8 ± 7.8	Sham acupuncture		The acupoint selection, treatment time and course of treatment were the same as those in the Acupuncture group.	
W He (37) 2012	China	30/30	18–54^c^	Auricular acupuncture	4	After 3 days, the contralateral ear was replaced. Each acupoint was pressed 10 s × 3 times a day by yourself (total 30 s/acupoint/day).	BMI, WC
				Usual care		Lifestyle counseling, including diet control and exercise instruction	
Darbandi (32) 2014	Iranian	20/20	39.00 ± 1.80	Auricular acupuncture	6	Seed into the acupoints on both ears in each treatment and kept on the ear for 3 days. All participants were requested to apply pressure to the auricular points before eating. Two treatment sessions per week for a total of 6 weeks.	BMI, WC
			37.90 ± 1.50	Sham auricular acupuncture		Received sham auricular acupuncture using placebo plasters, The treatment procedure was the same as the Auricular acupuncture group.	
Yeo S (35) 2014	Korea	22/15	34.70 ± 11.90	Auricular acupuncture	8	Unilateral auricular acupuncture was implanted once a week, alternately between the left and right ears, for 8 weeks. The auricular needle was embedded at a depth of 2 mm, and “push-pin” auricular needle was used and affixed to the tape for 1 week.	BMI, WC
			42.70 ± 10.20	sham Auricular acupuncture		Sham acupuncture was applied to the five acupoints in treatment groupI, alternating ears every week for 8 weeks.	
Chen IJ (50) 2018	China (Taiwan)	40/40	39.90 ± 9.80	Acupoint catgut embedding	12	Once a week for a total of 6 sessions.	BMI, WC
			43.70 ± 9.30	Sham embedding		Acupuncture at the same acupoints without thread embedding (sham embedding)	
DD Mao (58) 2018	China	38/36	38.16 ± 10.75	Manual therapy	12	Each site was scraped 20–30 times with the degree of scraping out. Once a week for 4 consecutive weeks as a cycle, a total of 3 cycles.	BMI, WC
			37.67 ± 10.34	Usual care		Lifestyle counseling.	
Suen L (34) 2019	China (Hong Kong)	21/19	50.52 ± 10.58	Auricular acupuncture	8	Change the tapes every 3 to 4 days, Subjects were requested to apply pressure 20 times using a constant rhythm to each point thrice per day, preferably within 30 min before meals.	BMI, WC
			47.58 ± 11.59	Usual care		Lifestyle counseling, including diet control and exercise instruction.	
ZX Li (46) 2019	China	30/30	35.92 ± 13.36	Acupuncture	12	On basis of the conventional treatment group, with the intervention given once every other day, needles retained for 30 min per session, and 18 sessions forming one complete course of treatment.	BMI, WC
			37.84 ± 13.90	Usual care		Lifestyle counseling, exercise instruction	
LS Chen (54) 2019	China (Macao)	28/23	34.43 ± 7.63	Acupoint catgut embedding	10	The treatment cycle was repeated every 10 days, and the therapeutic time continued for 10 weeks.	BMI, WC
			34.60 ± 5.21	Usual care		Lifestyle counseling, including diet control and exercise instruction.	
Kim KW (40) 2020	Korea	60/60	36.83 ± 8.79	Acupuncture	6	The needle was retained for 30 min after hand acupuncture to induce deqi. The electroacupuncture was connected to the device, and the stimulation was delivered at 2 mA, 25 Hz (25 min) + 60 Hz (5 min). The sessions were conducted twice a week for 6 weeks.	BMI, WC
			36.92 ± 7.93	Sham acupuncture		Non-penetrating sham needles were used at the same acupoints, The time and number of sessions were the same as in the intervention group.	
Y Zhang (56) 2020	China	43/41	43.89 ± 8.34	Manual therapy	5	The treatment was administered twice per week, with an interval of at least 2 days between sessions. Each session lasted 20 min, for a total duration of 5 weeks.	BMI, WC
			44.74 ± 7.69	Usual care		Lifestyle counseling	
W Zhou (44) 2020	China	45/45	18–45^c^	Acupuncture	8	Retain needle for 30 min, once every other day, 15 times per course; 3-day interval between courses, 2 courses total (suspended during menstruation).	BMI, WC
				Acupoint catgut embedding		Once a day, 3 times for a course of treatment. The interval between treatment sessions was 3 days before the next oneTreatment courses, 2 courses in total (menstrual period is suspended).	
YB Wang (65) 2021	China	22/22	62. 00 ± 5. 07	Traditional Chinese Exercises	12	Tai Chi exercises were performed four times a week for 90 min each time.	BMI
			59. 38 ± 4. 51	Usual care		Lifestyle counseling, No exercise intervention was performed.	
SF Wang (59) 2021	China	35/35	38.01 ± 7.94	Manual therapy	8	Treatment was given once weekly, with about 30 strokes per area,the course lasted 8 weeks, suspended during menstruation.	BMI, WC
			36.77 ± 8.24	Usual care		Lifestyle counseling	
L Dai (48) 2022	China	42/42	33.05 ± 6.60	Acupoint catgut embedding	11	PPDO absorbable suture was used and embedded into 4 acupoints (CV12, ST25, ST40, BL20). The treatment was given once every 10 days for a total of 8 times. After treatment, patients were asked to gently massage the acupoints for 2 min before and after meals daily.	BMI, WC
			36.17 ± 9.71	Sham embedding		For the SLAS arm, the treatment procedure was the same, except that no PPDO suture was in the embedding needle.	
H Wan (47) 2022	China	68/63	34 ± 4	Acupoint catgut embedding	12	The treatment was given once every 2 weeks for 12 weeks.	BMI, WC
				Sham embedding		The same type of embedding needle was used to perform acupuncture at the same depth (2 cm subcutaneous), but no thread was embedded, The time and number of sessions were the same as in the intervention group.	
Lima IG (45) 2022	Brazil	25/25	22.0 ± 2.29	Acupuncture	5	Ea (40 Hz) was applied to 7 acupoints for 40 min each time, 10 times in total.	BMI, WC
			22.0 ± 1.88	Usual care		Lifestyle counseling	
Y Wang (53) 2023	China	48/48	50 ± 4	Acupoint catgut embedding	8	The treatment was given once every 2 weeks for a total of 4 sessions for 8 weeks	BMI, WC
			49 ± 4	Usual care		Lifestyle counseling	
DW Zhang (63) 2023	China	34/34	19.46 ± 0.68	Traditional Chinese Exercises	8	7 sessions per week (including 5 teacher-led sessions and 2 sessions Practice by yourself), 45 min each time.	BMI, WC
			19.54 ± 0.73	Usual care		Lifestyle counseling	
Razzaghi M (39) 2023	Iranian	30/30	42.53 ± 9.84	Acupuncture	4	Laser irradiation was applied to each of the 16 relevant acupoints for 10 s per point. Treatment was administered three times per week for 4 consecutive weeks, totaling 12 sessions.	BMI, WC
			40.77 ± 7.87	Sham acupuncture		A sham laser device with no output was used on the same acupoints as the intervention group. The duration of irradiation and the number of treatment sessions were identical to those in the intervention group.	
YH Cui (43) 2023	China	54/54	33.08 ± 9.75	Acupuncture	8	On the basis of usual care, acupuncture was given to 12 acupoints, 5 days a week, once a day for 8 weeks	BMI, WC
			32.57 ± 9.84	Usual care		Lifestyle counseling	
WF Fan (36) 2024	China	13/11	32.50^a^ (27.25–39.5)^b^	Auricular acupuncture	4	The unilateral ear (selected acupoints including spleen point and endocrine point) was treated twice a day, 5 days a week, for a total of 4 weeks.	BMI, WC
			26^a^ (23.75–32)^b^	Usual care		Lifestyle counseling	
HL Luo (31) 2024	China	30/30	35.50^a^ (25.00–45.00)^b^	moxibustion	4	Moxibustion for 30 min, once every other day, 3 times as a course of treatment. The patients were treated for 4 consecutive courses.	BMI, WC
			35.00^a^ (28.50–46.00)^b^	Usual care		Dietary, psychological and behavioral guidance.	
Lam TF (41) 2024	China (Hong Kong)	84/84	46.80 ± 11.10	Acupuncture	8	Electroacupuncture treatment, 8 acupoints, 16 treatments, 2 times a week for 8 weeks.	BMI, WC
			48.20 ± 10.90	Sham acupuncture		Non-invasive sham acupuncture, same acupoint, no electrical stimulation	
YY Jin (55) 2024	China	41/41	40–60^c^	Acupoint catgut embedding	8	Every 2 weeks for 8 consecutive weeks.	BMI, WC
				Sham embedding		For the SLAS arm, the treatment procedure was the same, except that no absorbable surgical suture is inserted.	
LN Zhou (57) 2024	China	41/39	38^a^ (29–47.5)^b^	Manual therapy	8	A course of treatment was given 7 times, twice a week, with an interval of 3–4 days. A course of treatment was completed in 4 weeks for a total of 8 weeks, avoiding the menstrual period.	BMI, WC
			36^a^ (30–40)^b^	Usual care		Lifestyle counseling	
JM Chen (60) 2024	China	97/97	35.31 ± 7.96	Manual therapy	8	Treatment was given every 3 days (or 7-day delay in women) for 8 weeks.	BMI, WC
			36.62 ± 8.37	Usual care		Lifestyle counseling	
YJ Zhang (30) 2025	China	32/31	37.20 ± 10.50	Moxibustion	8	On the basis of Lifestyle counseling, moxibustion therapy was given, Each treatment lasted 30 min, and the treatment was given 3 times a week.	BMI, WC
			38.60 ± 11.60	Sham moxibustion		The acupoint selection, treatment time and course of treatment were the same as those in the moxibustion group.	
CW Fu (29) 2025	China	70/69	37.00^a^ (27.00–41.25)^b^	Moxibustion	8	On the basis of usual care, moxibustion therapy was given, 20 min each time, once every other day, 3 times a week.	BMI, WC
			33.00^a^ (27.50–39.50)^b^	Usual care		Lifestyle counseling, including diet control and exercise instruction.	
CF Zhang (38) 2025	China	19/19	31.42 ± 7.58	Auricular acupuncture	4	The treatment was given once in the morning and once in the evening, alternately in both ears, 30 min each time, 4 days a week, a total of 4 weeks.	BMI
			26^a^ (25–33)^b^	Usual care		Lifestyle counseling	
JR Wang (47) 2025	China	60/60	18–65^c^	Acupuncture	12	The needle was retained for 30 min three times a week for 12 weeks.	BMI, WC
				Usual care		Lifestyle counseling	
QW Yang (51) 2025	China	59/60	34.00 ± 12.00	Acupoint catgut embedding	12	The treatment was given every 2 weeks for a total of 6 sessions, and the total treatment period was 12 weeks.	BMI, WC
			35.00 ± 16.00	Sham embedding		Acupuncture at the same acupoints without thread embedding (sham embedding).	
HR Peng (52) 2025	China	31/30	34.59 ± 4.08	Acupoint catgut embedding	8	Catgut embedding was performed once a week, and two treatments were taken as a course of treatment.	BMI
				Acupuncture		Acupuncture was applied for 30 min, 5 times a week, 10 times as a course of treatment.	
LL Chen (61) 2025	China	33/31	31.94 ± 6.62	Manual therapy	4	The treatment was performed 5 times a week by pressing and kneading abdominal acupoints, transporting the abdomen, vibrating the abdomen, taking and kneading the belt vein, and kneading the points.	BMI, WC
			32.48 ± 5.58	Usual care		Lifestyle counseling	
Y Li (64) 2025	China	18/18	18–22^c^	Traditional Chinese Exercises	8	Received Baduanjin combined with resistance training intervention, specifically, the first 3 min of warm-up activities to achieve the effect of warm-up, then about 15 min of Baduanjin exercise, then about 18 min of resistance training with an interval of 1 min, and finally 3 min of stretching and relaxation.	BMI
				Usual care		Lifestyle counseling, including exercise instruction	
Y Yu (62) 2025	China	26/24	33.60 ± 7.40	Traditional Chinese Exercises	12	On the basis of the control group, the online interactive enhanced Baduanjin exercise was performed three times a week, 60 min each time, for 12 weeks.	BMI, WC
			38.20 ± 9.30	Usual care		Lifestyle counseling, they received three 60-min online health education sessions over 12 weeks.	

^a^Median age; ^b^Interquartile range of patients’ ages; ^c^Age range of patients; E: experimental intervention; C: control intervention; BMI: body mass index; WC: waist circumference; PPDO: poly-p-dioxanone; SLAS: sham long-term acupoint stimulation.

### 3.3 Risk of bias analysis

The results of the risk of bias analysis are provided in Supplementary Appendix 3. For random sequence generation, 31 studies were judged to have low risk of bias and 6 studies were judged to have an unclear risk of bias. For adequate allocation concealment, 18 studies showed unclear risk of bias, while 3 studies showed high risk of bias. Regarding blinding of participants and personnel, 24 studies showed high risk of bias and 13 studies showed low risk of bias. For blinding of outcome assessment, 3 studies showed high risk, 18 studies showed unclear risk, and 16 studies showed low risk of bias. In terms of incomplete outcome data, 30 studies were judged as low risk and 7 studies as unclear risk of bias. For selective reporting, 4 studies showed unclear risk of bias. For other biases, 14 studies showed unclear risk and 2 studies showed high risk of bias. Overall, 9(24.3%) studies were assessed as low risk of bias, 3(8.1%) studies as unclear risk, and 25(67.6%) studies as high risk of bias. The funnel plot was generally symmetrical. Egger’s test showed no publication bias for BMI and WC (*p* > 0.05; Supplementary Appendix 4).

### 3.4 Main findings per outcome

#### 3.4.1 BMI

Table 2 summarizes the network meta-analysis findings for BMI. Moxibustion ranked as the most effective intervention (SUCRA = 98.8%). Significant BMI reductions versus control were observed for moxibustion (SMD = −1.39, 95%CI: −1.97 to −0.81), manual therapy (SMD = −0.61, 95%CI: −0.94 to −0.28), and acupoint catgut embedding (SMD = −0.68, 95%CI: −1.27 to −0.09). Relative to placebo, significant benefits were also evident for moxibustion (SMD = −1.75, 95%CI: −2.68 to −0.83), manual therapy (SMD = −0.97, 95%CI: −1.76 to −0.18), acupoint catgut embedding (SMD = −1.04, 95%CI: −1.97 to −0.11), and auricular acupuncture (SMD = −0.52, 95%CI: −0.99 to −0.04). Based on the SUCRA, the descending order of efficacy was as follows: moxibustion (98.8%) > acupoint catgut embedding (70.1%) > manual therapy (65.7%) > auricular acupuncture (57.9%) > traditional Chinese exercises (48.4%) > acupuncture (41.6%) > control (13.4%) > placebo (4.1%).

**Table 2:** Result of network meta-analyses on non-pharmacological classes for BMI.

BMI							
**TCE** (SUCRA = 48.4%)	–	–	–	–	–	–	–
−0.78 (−1.63, 0.06)	**PL** (SUCRA = 4.1%)	–	–	–	–	–	–
**0.97 (0.24, 1.70)**	**1.75 (0.83, 2.68)**	**Mox (SUCRA = 98.8%)**	–	**–**	–	–	**–**
0.19 (−0.37, 0.74)	**0.97 (0.18, 1.76)**	**−0.78 (−1.45, −0.11)**	**MT** (SUCRA = 65.7%)	**–**	**–**	–	**–**
−0.42 (−0.87, 0.02)	0.36 (−0.36, 1.08)	**−1.39 (−1.97, −0.81)**	**−0.61 (−0.94, −0.28)**	**Control** (SUCRA = 13.4%)	**–**	**–**	–
−0.08 (−0.68, 0.53)	0.70 (−0.12, 1.53)	**−1.05 (−1.76, −0.33)**	−0.27 (−0.79, 0.26)	0.34 (−0.06, 0.75)	**Acu** (SUCRA = 41.6%)	**–**	–
0.26 (−0.48, 1.00)	**1.04 (0.11, 1.97)**	−0.71 (−1.54, 0.12)	0.07 (−0.61, 0.75)	**0.68 (0.09, 1.27)**	0.34 (−0.38, 1.05)	**ACE** (SUCRA = 70.1%)	**–**
−0.09 (−0.67, 0.48)	**0.52 (0.04, 0.99)**	0.17 (−0.45, 0.80)	0.12 (−0.80, 1.05)	0.47 (−0.46, 1.39)	−0.16 (−0.92, 0.59)	0.56 (−0.44, 1.56)	**AA** (SUCRA = 57.9%)

Treatments are reported in order of efficacy post-treatment with ranking according to surface under the cumulative ranking curves. Comparisons between treatments should be read from left to right, and the estimate is in the cell in common between the column-defining intervention and the row-defining intervention. Efficacy post-treatment values are given as mean overall change in symptoms (mean differences [MDs]); MDs of more than 0 favor the row-defining intervention. Data in parentheses represent 95% credible intervals. To obtain MDs for comparisons in the opposite direction, negative values should be converted into positive values, and vice versa. Significant results are set in boldface. PL, placebo therapy. Control, usual care. TCE, traditional Chinese exercises. Mox, moxibustion. MT, manual therapy. ACE, acupoint catgut embedding. AA, auricular acupuncture. Acu, acupuncture.

#### 3.4.2 WC

Supplementary Appendix 5 summarizes the network meta-analysis findings for WC. Moxibustion again ranked as the most effective intervention (SUCRA = 90.0%). Significant WC reductions versus control were observed for moxibustion (SMD = −1.48, 95%CI: −2.31 to −0.64), manual therapy (SMD = −0.82, 95%CI: −1.30 to −0.34), and placebo (SMD = −1.10, 95%CI: −2.16 to −0.04). Based on the SUCRA, the descending order of efficacy was as follows: moxibustion (90.0%) > placebo (72.9%) > auricular acupuncture (57.3%) > manual therapy (56.7%) > acupoint catgut embedding (47.1%) > acupuncture (39.2%) > traditional Chinese exercises (33.2%) > control (3.6%).

#### 3.4.3 Adverse events

Of the 37 included studies, 15 (35–38, 43, 44, 46, 47, 56–59, 61, 63–65) provided no description or reporting on operational safety, while the remaining 22 included safety assessments. Of these, 6 studies (29, 31, 32, 39, 43, 60) documented no adverse events. Observed adverse reactions mainly included subcutaneous ecchymosis, dizziness, and local pain or discomfort, primarily associated with acupoint catgut embedding (48–55), acupuncture (40–42, 45), and auricular acupoint therapy (33, 34). In addition, moxibustion (30) was linked to local redness and mild burns, and therapeutic exercise (62) was associated with muscle soreness and post-exercise fatigue. All adverse reactions resolved after symptomatic management, and no serious adverse events were reported in any study. None of the included studies used standardized adverse event assessment scales, nor did they provide detailed information such as severity grading of adverse events.

### 3.5 Subgroup analysis

Subgroup analyses were performed to explore whether intervention duration, treatment frequency, and sample size could moderate the treatment effects on BMI and WC, with results presented in Supplementary Appendix 6. Overall, for BMI, subgroup stratifications by sample size ≤60, intervention duration ≤4 weeks, and treatment frequency ≥5 times per week substantially reduced heterogeneity. Other subgroup stratifications did not substantially reduce heterogeneity, and I^2^ remained moderate to high across all models (I^2^ ≈ 49.9–88.5%). This indicates that these moderators explain only a limited proportion of between-study variation, and results should therefore be interpreted with caution. All subgroups showed statistically significant reductions in BMI and WC (all *p* < 0.05), indicating that these factors did not eliminate the overall treatment effect, though effect magnitudes varied.

### 3.6 Assessment of inconsistency

The global inconsistency test showed no significant inconsistency between direct and indirect evidence within the closed loops for BMI (*p* = 0.77) and WC (*p* = 0.71). In line with this, local inconsistency analysis based on the node-splitting method indicated no significant inconsistency across all pairwise comparisons (all *p* > 0.05). Detailed outcomes of the inconsistency tests are systematically presented in Supplementary Appendix 4.

### 3.7 Additional analysis

A sensitivity analysis was performed to assess the robustness of the pooled results, which demonstrated that the network meta-analysis findings were not substantially altered (Supplementary Appendix 7). Meta-regression analyses revealed that most covariates (Supplementary Appendix 6) did not significantly modify the relative treatment effects. The certainty of evidence for these interventions in improving BMI and WC was moderate to very low. Detailed CINeMA results for all comparisons are presented in Supplementary Appendix 9.

## 4 Discussion

To our knowledge, this is the first network meta-analysis comparing the relative effectiveness of multiple TCM non-pharmacological interventions for simple obesity. The study integrated data from 37 RCTs involving 2,832 participants and 6 distinct TCM non-pharmacological interventions. The NMA results suggested that moxibustion may offer a relative advantage in reducing both BMI and WC. Acupoint catgut embedding was also associated with larger BMI reduction. According to SUCRA, the top three interventions for BMI reduction were moxibustion, acupoint catgut embedding, and manual therapy. For WC, moxibustion also showed greater effects, with placebo and auricular acupuncture also yielded notable reductions. Overall, different TCM non-pharmacological interventions may have varying effects on obesity-related outcomes. Moxibustion appeared to perform relatively well for both BMI and WC.

According to the CINeMA framework, the quality of evidence for moxibustion in reducing BMI and WC was rated as moderate and low, respectively. Nevertheless, the effect sizes for BMI (SMD = −1.39, 95% CI: −1.97 to −0.81, SUCRA = 98.8%) and WC (SMD = −1.48, 95% CI: −2.31 to −0.64, SUCRA = 90.0%) indicated potentially favorable efficacy. However, these findings should be interpreted cautiously given the limitations in evidence quality. Taken together, the relatively large effect sizes and stable ranking patterns suggest that moxibustion may be a promising non-pharmacological intervention for simple obesity management, without implying definitive superiority. This result is consistent with previous evidence supporting the potential benefits of moxibustion for simple obesity, indicating favorable changes in health-related outcomes (66, 67). As a characteristic therapy in TCM, moxibustion is a non-invasive, well-tolerated and easily implemented non-pharmacological intervention that has gained increasing recognition in obesity management, while avoiding adverse drug reactions and risks associated with invasive procedures. Experimental studies in mice have shown that moxibustion promotes the browning of white adipose tissue, thereby ameliorating obesity and related metabolic abnormalities (68). It also significantly activates the hypothalamic cAMP/PKA/CREB signaling pathway, upregulates the expression of thermogenic genes such as *UCP1*, *Prdm16*, and *PGC-1α*, and induces the conversion of white fat to brown/beige fat, thereby increasing energy expenditure and reducing body weight. In addition, a recent review has systematically summarized the mechanisms by which moxibustion treats obesity by promoting white fat browning (69). The warm stimulation from moxibustion on specific acupoints may regulate meridian circulation and zang-fu organ function, which may contribute to reductions in body weight and blood lipids with no major reported adverse events. Furthermore, moxibustion at CV12 (Zhongwan) can significantly downregulate serum leptin levels, upregulate the expression of leptin receptors in the hypothalamus, and restore leptin sensitivity, thereby inhibiting appetite and reducing food intake (70).

It is worth noting that in this study, placebo ranked second in the SUCRA ranking for WC reduction. In obesity research, placebo interventions are often accompanied by dietary and exercise guidance. Systematic reviews have indicated the presence of a notable placebo effect, with effect sizes varying across outcome measures (71). Furthermore, WC is likely to be more susceptible to lifestyle co-interventions, short-term behavioral modifications, and measurement errors than BMI (72); consequently, the SUCRA ranking tends to shift upward when sample sizes are limited and effect sizes are similar. Therefore, it is inappropriate to conclude that the placebo is superior to most TCM treatments for reducing WC based solely on this ranking. Instead, such findings should be interpreted in conjunction with effect sizes, 95% CIs, heterogeneity, and evidence quality.

In addition to moxibustion therapy, acupoint catgut embedding also showed potentially beneficial effects on improving BMI and WC. Acupoint catgut embedding is a modified acupoint stimulation technique derived from traditional acupuncture, representing an innovative integration of classical acupuncture theory and modern biomedical technology. By implanting absorbable surgical sutures into specific acupoints, this modality produces sustained, mild, low-intensity mechanical stimulation to achieve prolonged therapeutic effects. In the present study, acupoint catgut embedding ranked second for improving BMI (SMD = −0.68, 95% CI: −1.27 to −0.09, SUCRA = 70.01%). These results are consistent with previous studies suggesting that acupoint catgut embedding may help suppress appetite in individuals with obesity, may help ameliorate endocrine imbalance, and may reduce BMI via modulating the sympathetic–adrenal cortex and hypothalamic–pituitary–adrenal cortex systems (73, 74). However, there is still a lack of high-quality and large-sample studies that directly compare moxibustion and simple thread-embedding, and the above explanation needs to be verified.

Despite this statistical stability, we observed moderate-to-high heterogeneity. Although meta-regression analyses revealed that most covariates did not significantly modify the relative treatment effects, our subgroup analyses demonstrated that treatment duration and intervention frequency significantly moderated the efficacy of TCM non-pharmacological interventions for simple obesity. Specifically, an optimal therapeutic window of 4–8 weeks yielded more favorable BMI reductions than shorter or longer durations, while interventions of shorter duration demonstrated the most robust effects on WC. Additionally, interventions delivered at a moderate weekly frequency (three to four sessions per week) were associated with the most pronounced reductions in WC. Trials with moderate sample sizes (60–100 participants) showed the largest effect sizes, whereas larger multicenter trials exhibited attenuated effects, likely due to greater heterogeneity in intervention delivery, acupoint selection, operator proficiency, and patient characteristics. This reveals a notable efficacy–effectiveness gap: while TCM non-pharmacological interventions perform well in controlled settings, their real-world effectiveness remains insufficiently defined. Future large-scale trials should prioritize intervention standardization, operator training, and fidelity monitoring to bridge this efficacy–effectiveness gap.

### 4.1 Strengths and limitations

This study has several strengths: (1) This is the first NMA to systematically classify and compare various TCM non-pharmacological interventions for simple obesity; (2) Only RCTs were included, ensuring a rigorous study design. However, this study also has several limitations. First, most included studies had short-to-medium follow-up durations (≤8 weeks), precluding assessment of long-term weight maintenance and effect sustainability. Second, substantial heterogeneity persisted in several comparisons despite subgroup analyses, likely attributable to variations in acupoint selection, stimulation parameters, and co-interventions. Third, the majority of trials were conducted in Chinese populations, limiting generalizability to other ethnic groups. Fourth, risk of bias assessment revealed that approximately 60% of studies were rated as having high or unclear risk, predominantly due to inadequate blinding—an inherent challenge in non-pharmacological intervention research. Finally, although the classification of manual therapy protocols in this study was based on previous systematic reviews or other non-pharmacological interventions (75, 76), integrating Gua sha, massage, and cupping therapy into a single aggregate node may have contributed to result heterogeneity. Therefore, the SUCRA rankings presented in this study should be interpreted as probabilistic treatment rankings within the network rather than definitive evidence of clinical superiority.

### 4.2 Implications for clinical practice and future research

Our findings carry certain reference value for clinical practice and research. For adults with simple obesity whose main treatment goals are improvement in body mass index and waist circumference, moxibustion may be considered as a non-pharmacological intervention option. Acupoint catgut embedding may also serve as an alternative, particularly for individuals who prefer fewer treatment sessions. However, given the methodological limitations, between-study heterogeneity, and the overall uncertainty of the current evidence, these interventions should still be applied with caution in clinical practice. Specific treatment decisions should be made after considering the patient’s individual condition, preferences, treatment accessibility, and the practitioner’s experience. Subgroup analyses further suggested that a treatment course of 4 to 8 weeks with 3 to 4 sessions per week may be associated with better outcomes. However, these findings remain exploratory at present and still require confirmation in well-designed prospective studies. For future research, additional high-quality, multicenter randomized controlled trials with larger sample sizes are needed to compare the effectiveness of different TCM non-pharmacological interventions and to evaluate their long-term effects on weight control.

## 5 Conclusion

This network meta-analysis provides a quantitative synthesis of the available evidence on non-pharmacological TCM treatments for adults with simple obesity. The results suggest that there are certain differences in the effects of different TCM non-drug therapies included in this study on improving body mass index and waist circumference, and moxibustion may have a relative advantage in the performance of related outcome indicators. These findings should be interpreted with caution given the uncertainty of the available evidence and the limitations of the included studies. In the future, larger sample size and higher quality randomized controlled trials are needed to verify the findings of this study and further clarify the clinical position of these therapies in the comprehensive management of simple obesity.

## Data Availability

The original contributions presented in the study are included in the article/Supplementary material, further inquiries can be directed to the corresponding authors.
