# Supplementary material for: Effects of traditional Chinese medicine non-pharmacological intervention in patients with simple obesity: a systematic review and network meta-analysis
Source: Front Med (Lausanne). 2026 May 8;13:1815757. doi: 10.3389/fmed.2026.1815757 (PMC13194107; doi:10.3389/fmed.2026.1815757)
Supplement: Supplementary file 1 [file Data_Sheet_1.DOCX]

***Supplementary Material***

[Appendix 1: PRISMA NMA Checklist of Items to Include When Reporting A Systematic Review Involving a Network Meta-analysis 2](#_Toc226722421)

[Appendix 2: Search strategy 7](#_Toc226722422)

[Appendix 3 Results of risk of bias 9](#_Toc226722423)

[Appendix 4 Funnel Plots and Inconsistency Assessment 12](#_Toc226722424)

[Appendix 4-1 BMI funnel plots 12](#_Toc226722425)

[Appendix 4-2 BMI. Assessment of inconsistency. 13](#_Toc226722426)

[Appendix 4-3 WC funnel plots 14](#_Toc226722427)

[Appendix 4-4 WC. Assessment of inconsistency. 15](#_Toc226722428)

[Appendix 5 Result of network meta-analyses on non-pharmacological classes for WC 16](#_Toc226722429)

[Appendix 6 Subgroup Analyses for BMI, WC 18](#_Toc226722430)

[Appendix 7 Sensitivity analysis for BMI, WC 20](#_Toc226722431)

[Appendix 8 BMI、WC Forest Plot 22](#_Toc226722432)

[Appendix 9 Grade of evidence assessment using the CINeMA (Confidence in Network Meta 23](#_Toc226722433)

[Appendix 9-1 CINEMA Assessments for BMI 23](#_Toc226722434)

[Appendix 9-2 CINEMA Assessments for WC 24](#_Toc226722435)

# Appendix 1: PRISMA NMA Checklist of Items to Include When Reporting A Systematic Review Involving a Network Meta-analysis

| **Section/Topic** | **Item #** | **Checklist Item** | **Reported on Page #** |
| --- | --- | --- | --- |
| **TITLE** |  |  |  |
| Title | 1 | Identify the report as a systematic review *incorporating a network meta-analysis (or related form of meta-analysis).* | ***P1（Title page）*** |
|  |  |  |  |
| **ABSTRACT** |  |  |  |
| Structured summary | 2 | Provide a structured summary including, as applicable:  **Background:** main objectives  **Methods:** data sources; study eligibility criteria, participants, and interventions; study appraisal; and *synthesis methods, such as network meta-analysis.*  **Results:** number of studies and participants identified; summary estimates with corresponding confidence/credible intervals; *treatment rankings may also be discussed. Authors may choose to summarize pairwise comparisons against a chosen treatment included in their analyses for brevity.*  **Discussion/Conclusions:** limitations; conclusions and implications of findings.  **Other:** primary source of funding; systematic review registration number with registry name. | P1（Abstract） |
|  |  |  |  |
| **INTRODUCTION** |  |  |  |
| Rationale | 3 | Describe the rationale for the review in the context of what is already known*, including mention of why a network meta-analysis has been conducted.* | ***P2-P3（Introduction - Rationale）*** |
| Objectives | 4 | Provide an explicit statement of questions being addressed, with reference to participants, interventions, comparisons, outcomes, and study design (PICOS). | P3（Introduction - Objectives） |
|  |  |  |  |
| **METHODS** |  |  |  |
| Protocol and registration | 5 | Indicate whether a review protocol exists and if and where it can be accessed (e.g., Web address); and, if available, provide registration information, including registration number. | P3（Methods - Registration） |
| Eligibility criteria | 6 | Specify study characteristics (e.g., PICOS, length of follow-up) and report characteristics (e.g., years considered, language, publication status) used as criteria for eligibility, giving rationale. *Clearly describe eligible treatments included in the treatment network, and note whether any have been clustered or merged into the same node (with justification).* | ***P3-P4（Methods - Eligibility criteria）*** |
| Information sources | 7 | Describe all information sources (e.g., databases with dates of coverage, contact with study authors to identify additional studies) in the search and date last searched. | P3（Methods - Search strategy） |
| Search | 8 | Present full electronic search strategy for at least one database, including any limits used, such that it could be repeated. | P3（Methods - Search strategy）, Supplementary Appendix 2 |
| Study selection | 9 | State the process for selecting studies (i.e., screening, eligibility, included in systematic review, and, if applicable, included in the meta-analysis). | P4（Methods - Study selection） |
| Data collection process | 10 | Describe method of data extraction from reports (e.g., piloted forms, independently, in duplicate) and any processes for obtaining and confirming data from investigators. | P4（Methods - Data extraction） |
| Data items | 11 | List and define all variables for which data were sought (e.g., PICOS, funding sources) and any assumptions and simplifications made. | P4（Methods - Data extraction） |
| **Geometry of the network** | **S1** | Describe methods used to explore the geometry of the treatment network under study and potential biases related to it. This should include how the evidence base has been graphically summarized for presentation, and what characteristics were compiled and used to describe the evidence base to readers. | ***P5（Methods - Data analysis）*** |
| Risk of bias within individual studies | 12 | Describe methods used for assessing risk of bias of individual studies (including specification of whether this was done at the study or outcome level), and how this information is to be used in any data synthesis. | P4（Methods - Quality and certainty of evidence assessment） |
| Summary measures | 13 | State the principal summary measures (e.g., risk ratio, difference in means). *Also describe the use of additional summary measures assessed, such as treatment rankings and surface under the cumulative ranking curve (SUCRA) values, as well as modified approaches used to present summary findings from meta-analyses.* | P5（Methods - Data analysis） |
| Planned methods of analysis | 14 | Describe the methods of handling data and combining results of studies for each network meta-analysis. This should include, but not be limited to:   - *Handling of multi-arm trials;* - *Selection of variance structure;* - *Selection of prior distributions in Bayesian analyses; and* - *Assessment of model fit.* | P5（Methods - Data analysis） |
| **Assessment of Inconsistency** | **S2** | Describe the statistical methods used to evaluate the agreement of direct and indirect evidence in the treatment network(s) studied. Describe efforts taken to address its presence when found. | P5（Methods - Data analysis） |
| Risk of bias across studies | 15 | Specify any assessment of risk of bias that may affect the cumulative evidence (e.g., publication bias, selective reporting within studies). | **P5（Methods - Data analysis）** |
| Additional analyses | 16 | Describe methods of additional analyses if done, indicating which were pre-specified. This may include, but not be limited to, the following:   - Sensitivity or subgroup analyses; - Meta-regression analyses; - *Alternative formulations of the treatment network; and* - *Use of alternative prior distributions for Bayesian analyses (if applicable).* | ***P5（Methods - Data analysis）*** |
|  |  |  |  |
| **RESULTS†** |  |  |  |
| Study selection | 17 | Give numbers of studies screened, assessed for eligibility, and included in the review, with reasons for exclusions at each stage, ideally with a flow diagram. | P4（Results - Literature selection）, Figure 1 |
| **Presentation of network structure** | **S3** | Provide a network graph of the included studies to enable visualization of the geometry of the treatment network. | P5-P6***（Results - Characteristics of the included studies）, Figure 2*** |
| **Summary of network geometry** | **S4** | Provide a brief overview of characteristics of the treatment network. This may include commentary on the abundance of trials and randomized patients for the different interventions and pairwise comparisons in the network, gaps of evidence in the treatment network, and potential biases reflected by the network structure. | ***P5（Results - Characteristics of the included studies）、P6（Results - Network meta-analysis）*** |
| Study characteristics | 18 | For each study, present characteristics for which data were extracted (e.g., study size, PICOS, follow-up period) and provide the citations. | P5-P6（Results - Characteristics of the included studies）, Table 1 |
| Risk of bias within studies | 19 | Present data on risk of bias of each study and, if available, any outcome level assessment. | P6（Results - Risk of Bias Analysis）, Supplementary Appendix 3 |
| Results of individual studies | 20 | For all outcomes considered (benefits or harms), present, for each study: 1) simple summary data for each intervention group, and 2) effect estimates and confidence intervals. *Modified approaches may be needed to deal with information from larger networks.* | ***P5-P6（Results - Network meta-analysis）, Supplementary Appendices*** |
| Synthesis of results | 21 | Present results of each meta-analysis done, including confidence/credible intervals. *In larger networks, authors may focus on comparisons versus a particular comparator (e.g. placebo or standard care), with full findings presented in an appendix. League tables and forest plots may be considered to summarize pairwise comparisons.* If additional summary measures were explored (such as treatment rankings), these should also be presented. | ***P6（Results - Network meta-analysis）, Table 2, Supplementary Appendix 4,5*** |
| **Exploration for inconsistency** | **S5** | Describe results from investigations of inconsistency. This may include such information as measures of model fit to compare consistency and inconsistency models, *P* values from statistical tests, or summary of inconsistency estimates from different parts of the treatment network. | ***P7（Results - Assessment of inconsistency）, Supplementary Appendix 4*** |
| Risk of bias across studies | 22 | Present results of any assessment of risk of bias across studies for the evidence base being studied. | ***P6（Results - Risk of Bias Analysis）Supplementary Appendix 4*** |
| Results of additional analyses | 23 | Give results of additional analyses, if done (e.g., sensitivity or subgroup analyses, meta-regression analyses*, alternative network geometries studied, alternative choice of prior distributions for Bayesian analyses,* and so forth). | ***P7（Results - Subgroup analysis）, Supplementary Appendix 6,7*** |
|  |  |  |  |
| **DISCUSSION** |  |  |  |
| Summary of evidence | 24 | Summarize the main findings, including the strength of evidence for each main outcome; consider their relevance to key groups (e.g., healthcare providers, users, and policy-makers). | P7-P9（Discussion） |
| Limitations | 25 | Discuss limitations at study and outcome level (e.g., risk of bias), and at review level (e.g., incomplete retrieval of identified research, reporting bias). *Comment on the validity of the assumptions, such as transitivity and consistency. Comment on any concerns regarding network geometry (e.g., avoidance of certain comparisons).* | P9-P10（Discussion - Strengths and limitations） |
| Conclusions | 26 | Provide a general interpretation of the results in the context of other evidence, and implications for future research. | P10（Discussion-Conclusion） |
|  |  |  |  |
| **FUNDING** |  |  |  |
| Funding | 27 | Describe sources of funding for the systematic review and other support (e.g., supply of data); role of funders for the systematic review. This should also include information regarding whether funding has been received from manufacturers of treatments in the network and/or whether some of the authors are content experts with professional conflicts of interest that could affect use of treatments in the network. | ***P10（Sources of funding; Ethical approval & Consent）*** |

PICOS = population, intervention, comparators, outcomes, study design.

* Text in italics indicateS wording specific to reporting of network meta-analyses that has been added to guidance from the PRISMA statement.

† Authors may wish to plan for use of appendices to present all relevant information in full detail for items in this section.

# Appendix 2: Search strategy

**Pubmed**

#1 Search (adiposity[MeSH Terms]) OR (obesity[MeSH Terms])

#2 Search (((((weight loss[MeSH Terms]) OR (body weight[MeSH Terms])) OR (overweight[Title/Abstract])) OR (obesity[Title/Abstract])) OR (fat[Title/Abstract])) OR (weight reduction[Title/Abstract])

#3 #1 OR #2

#4 Search "medicine, chinese traditional"[MeSH Terms]

#5 Search (((traditional chinese medicine[Title/Abstract]) OR (Chinese Traditional Medicine[Title/Abstract])) OR (herbal medicine[Title/Abstract])) OR (Chinese herbal medicine[Title/Abstract])

#6 Search ((acupuncture[MeSH Terms]) OR (acupuncture therapy[MeSH Terms])) OR (acupuncture, ear[MeSH Terms])

#7 Search ((((((((((((((acupunctural[Title/Abstract]) OR (acupuncture[Title/Abstract])) OR (acupuncture therapy[Title/Abstract])) OR (acupunctures[Title/Abstract])) OR (acupunctured[Title/Abstract])) OR (acupuncturing[Title/Abstract])) OR (pharmacopuncture[Title/Abstract])) OR (pharmacopunctures[Title/Abstract])) OR (acupuncture treatment[Title/Abstract])) OR (acupuncture treatments[Title/Abstract])) OR (treatment, acupuncture[Title/Abstract])) OR (therapy, acupuncture[Title/Abstract])) OR (trigger point[Title/Abstract])) OR (acupuncture Point[Title/Abstract])) OR (point, acupuncture[Title/Abstract])

#8 Search "electroacupuncture"[MeSH Terms]

#9 Search ((((((electroacupuncture[Title/Abstract]) OR (electroacupuncturing[Title/Abstract])) OR (acup*[Title/Abstract])) OR (transcutanclus[Title/Abstract])) OR (acupoints[Title/Abstract])) OR (acupoint[Title/Abstract])) OR (electro-acupuncture[Title/Abstract])

#10 Search (((((needles[MeSH Terms]) OR (needles[Title/Abstract])) OR (needled[Title/Abstract])) OR (needle[Title/Abstract])) OR (needling[Title/Abstract])) OR (needlings[Title/Abstract])

#11 Search "acupoint catgut embedding"[Title/Abstract]

#12 Search (moxibustion[MeSH Terms]) OR (moxibustion[Title/Abstract])

#13 Search ((Cupping therapy[Title/Abstract]) OR (baguan[Title/Abstract])) OR (Gua Sha therapy[Title/Abstract])

#14 Search (Tai Ji[MeSH Terms]) OR (Qigong[MeSH Terms])

#15 Search ((((((((((((((((((((baduanjin[Title/Abstract]) OR (Baduanjin exercise[Title/Abstract])) OR (eight section brocade*[Title/Abstract])) OR (eight trigrams boxing[Title/Abstract])) OR (Yi Jinjing[Title/Abstract])) OR (Yijinjing[Title/Abstract])) OR (Yijinjing exercise[Title/Abstract])) OR (Tai Ji Quan[Title/Abstract])) OR (Tai chi[Title/Abstract])) OR (Tai ji[Title/Abstract])) OR (Taijiquan[Title/Abstract])) OR (five-animal exercises[Title/Abstract])) OR (Five Animals[Title/Abstract])) OR (five animals exercise[Title/Abstract])) OR (Five-Bird Game[Title/Abstract])) OR (six-character formula[Title/Abstract])) OR (qigong[Title/Abstract])) OR (Ch'i Kung[Title/Abstract])) OR (Traditional Chinese medicine exercises[Title/Abstract])) OR (Traditional Chinese exercises[Title/Abstract])) OR (traditional chinese sports[Title/Abstract])

#16 #4 or #5 or #6 or #7 or #8 or #9 or #10 or #11 or #12 or #13 or #14 or #15

#17 #3 and #16

# Appendix 3 Results of risk of bias

| **Study** | **Adequate sequence generation** | **Adequate allocation concealment** | **Blinding** | | **Incomplete outcome data addressed** | **Free of selective reporting** | **Other bias** | **overall RoB** |
| --- | --- | --- | --- | --- | --- | --- | --- | --- |
|  |  |  | **participant and personnel** | **outcome assessment** |  |  |  |  |
| **CW Fu**  2025 | low risk | low risk | high risk | unclear risk | low risk | low risk | low risk | high risk |
| **YJ Zhang**  **2025** | low risk | low risk | low risk | low risk | low risk | low risk | low risk | low risk |
| **HL Luo**  **2024** | low risk | unclear risk | high risk | low risk | unclear risk | low risk | low risk | high risk |
| **Darbandi**  **2014** | low risk | unclear risk | low risk | low risk | low risk | low risk | unclear risk | unclear risk |
| **Lien CY**  **2012** | low risk | low risk | low risk | low risk | unclear risk | low risk | low risk | unclear risk |
| **Suen L**  **2019** | unclear risk | unclear risk | high risk | high risk | low risk | unclear risk | unclear risk | high risk |
| **Güçel F**  **2012** | low risk | unclear risk | low risk | unclear risk | low risk | low risk | unclear risk | unclear risk |
| **Yeo S**  **2014** | low risk | low risk | low risk | high risk | unclear risk | unclear risk | unclear risk | high risk |
| **He W**  **2012** | unclear risk | unclear risk | high risk | low risk | low risk | low risk | unclear risk | high risk |
| **CF Zhang**  **2025** | low risk | unclear risk | high risk | unclear risk | low risk | low risk | unclear risk | high risk |
| **WF Fan**  **2024** | low risk | unclear risk | high risk | unclear risk | low risk | low risk | low risk | high risk |
| **Chen IJ**  **2018** | low risk | unclear risk | high risk | unclear risk | low risk | low risk | unclear risk | high risk |
| **QW Yang**  **2025** | low risk | low risk | low risk | low risk | low risk | low risk | low risk | low risk |
| **HR Peng**  **2025** | low risk | unclear risk | high risk | unclear risk | unclear risk | unclear risk | unclear risk | high risk |
| **Razzaghi M**  **2023** | low risk | low risk | low risk | low risk | low risk | low risk | low risk | low risk |
| **Kim KW**  **2020** | low risk | low risk | low risk | low risk | low risk | low risk | low risk | low risk |
| **H Wan**  **2022** | low risk | low risk | low risk | low risk | low risk | low risk | low risk | low risk |
| **L Dai**  **2022** | low risk | low risk | low risk | low risk | low risk | low risk | low risk | low risk |
| **Lam TF**  **2024** | low risk | low risk | low risk | low risk | low risk | low risk | low risk | low risk |
| **YH Cui**  **2023** | unclear risk | unclear risk | high risk | high risk | low risk | low risk | high risk | high risk |
| **Y Zhang**  **2020** | low risk | unclear risk | high risk | unclear risk | unclear risk | low risk | unclear risk | high risk |
| **SF Wang**  **2021** | low risk | unclear risk | high risk | unclear risk | low risk | low risk | low risk | high risk |
| **DD Mao**  **2018** | low risk | unclear risk | high risk | unclear risk | low risk | low risk | low risk | high risk |
| **LL Chen**  **2025** | low risk | unclear risk | high risk | low risk | low risk | low risk | low risk | high risk |
| **JM Chen**  **2024** | low risk | unclear risk | high risk | unclear risk | low risk | low risk | low risk | high risk |
| **Y Wang**  **2023** | low risk | low risk | high risk | unclear risk | low risk | low risk | low risk | high risk |
| **JR Wang**  **2025** | low risk | high risk | high risk | unclear risk | unclear risk | low risk | unclear risk | high risk |
| **Yu Y**  **2025** | low risk | low risk | high risk | unclear risk | low risk | low risk | low risk | high risk |
| **YB Wang**  **2021** | unclear risk | unclear risk | high risk | unclear risk | unclear risk | low risk | unclear risk | high risk |
| **DW Zhang**  **2023** | low risk | high risk | high risk | low risk | low risk | low risk | unclear risk | high risk |
| **W Zhou**  **2020** | unclear risk | low risk | high risk | unclear risk | low risk | low risk | high risk | high risk |
| **ZX Li**  **2019** | low risk | unclear risk | high risk | unclear risk | low risk | unclear risk | low risk | high risk |
| **LN Zhou**  **2024** | low risk | unclear risk | high risk | unclear risk | low risk | low risk | low risk | high risk |
| **Lima IG**  **2022** | low risk | low risk | high risk | low risk | low risk | low risk | unclear risk | high risk |
| **Y Li**  **2025** | low risk | low risk | low risk | low risk | low risk | low risk | low risk | low risk |
| **LS Chen**  **2019** | unclear risk | high risk | high risk | unclear risk | low risk | low risk | unclear risk | high risk |
| **YY Jin**  **2024** | low risk | low risk | low risk | low risk | low risk | low risk | low risk | low risk |

# Appendix 4 Funnel Plots and Inconsistency Assessment

## Appendix 4-1 BMI funnel plots


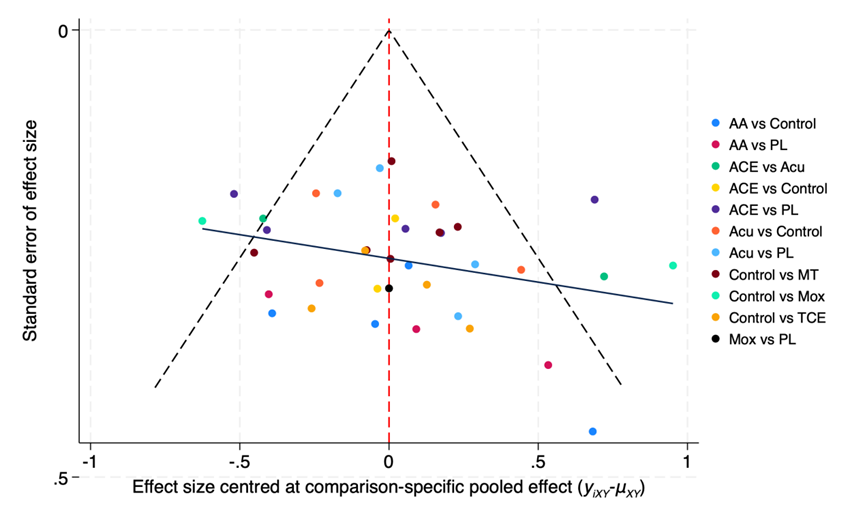


Egger’s test P = 0.9606

##
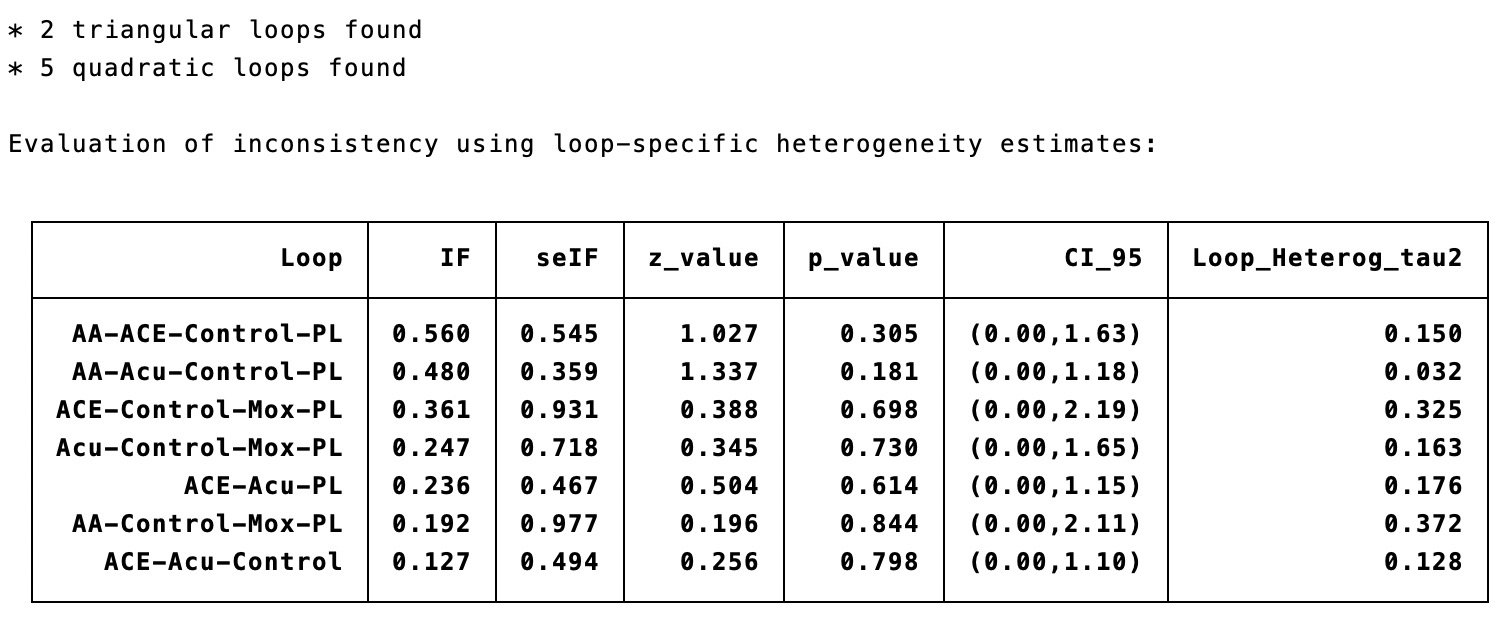

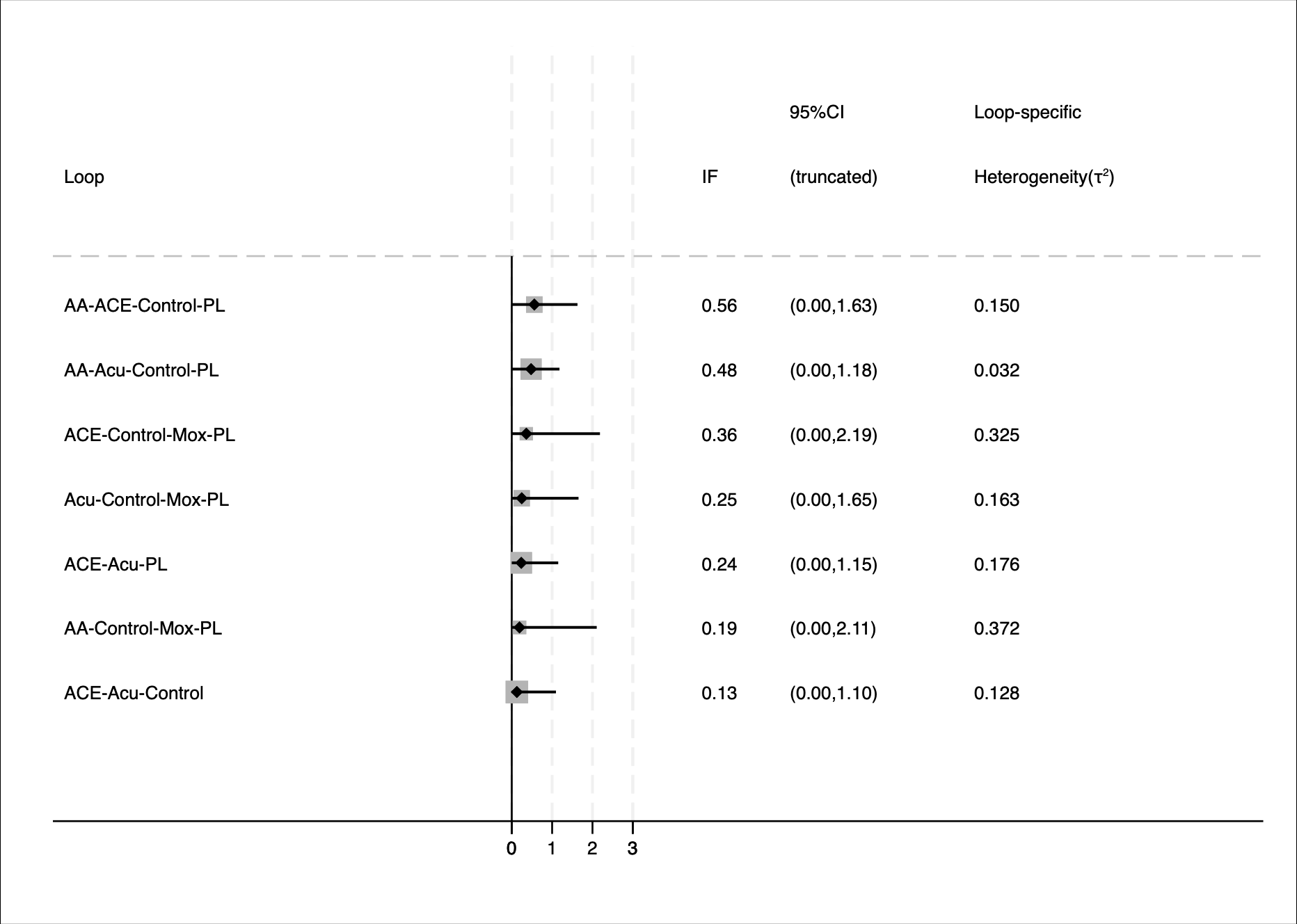
Appendix 4-2 BMI. Assessment of inconsistency.

## Appendix 4-3 WC funnel plots


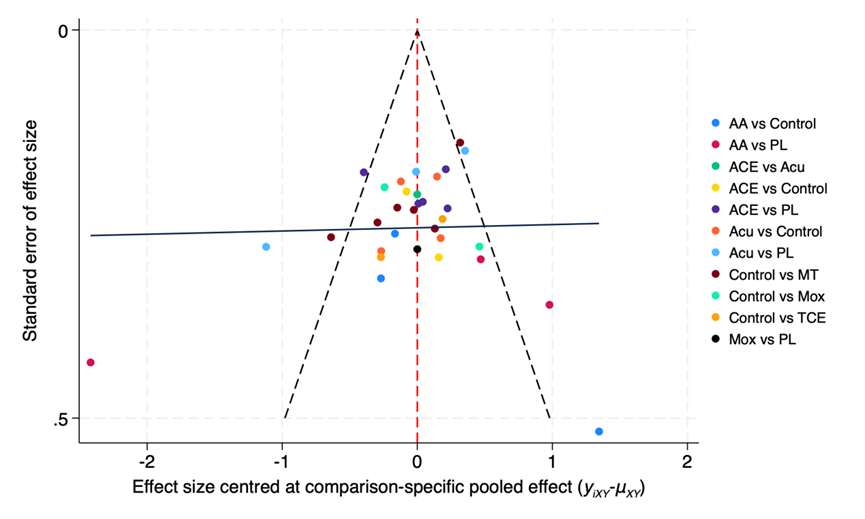


Egger’s test P = 0.9391

## Appendix 4-4 WC. Assessment of inconsistency.

**
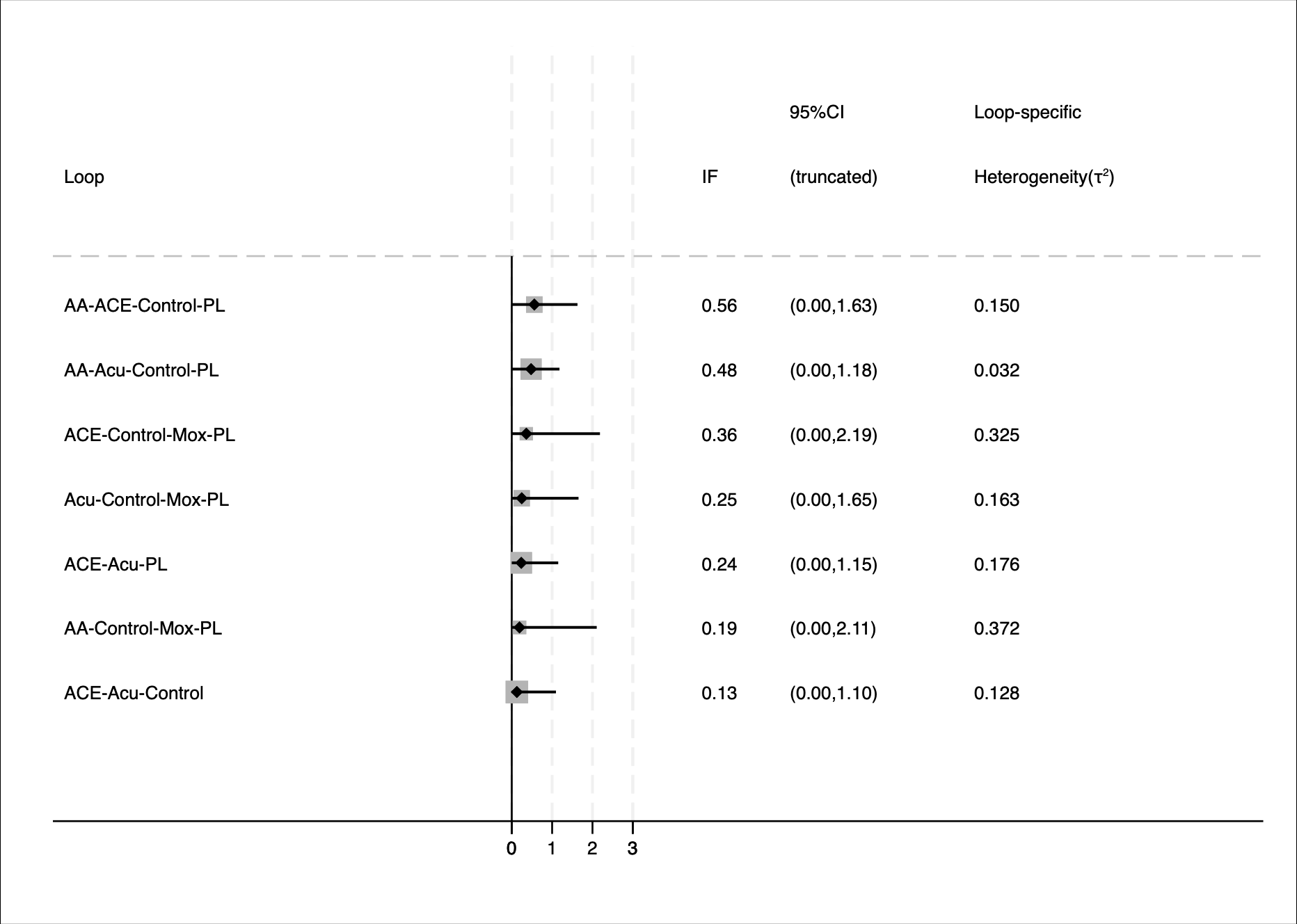
**


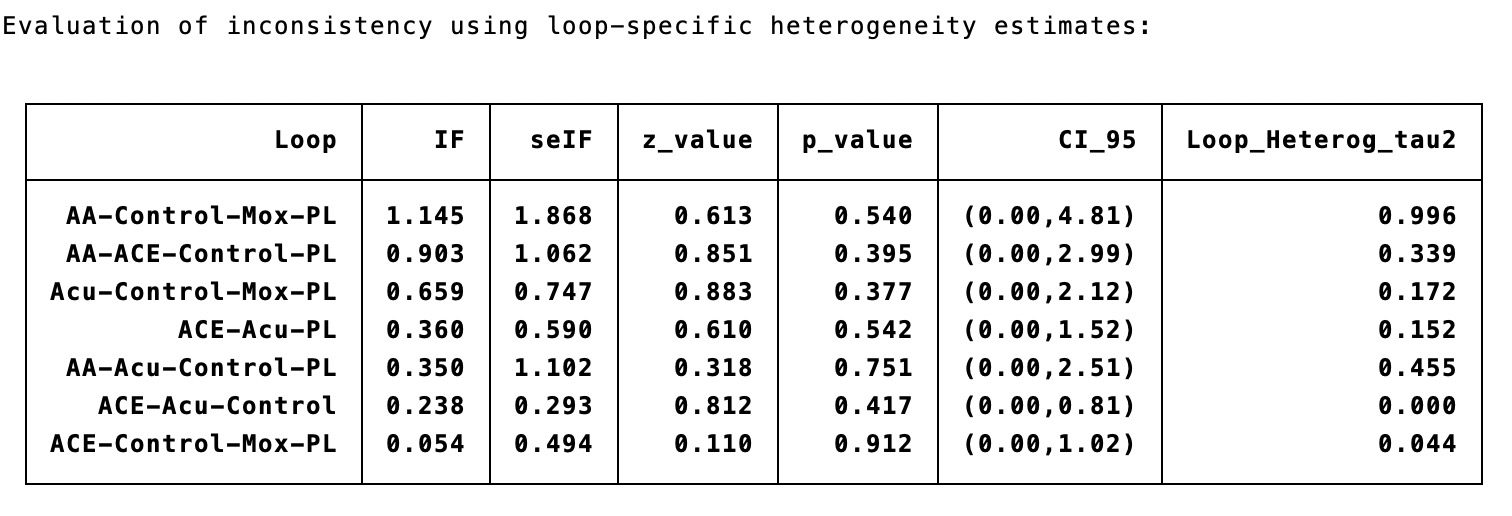


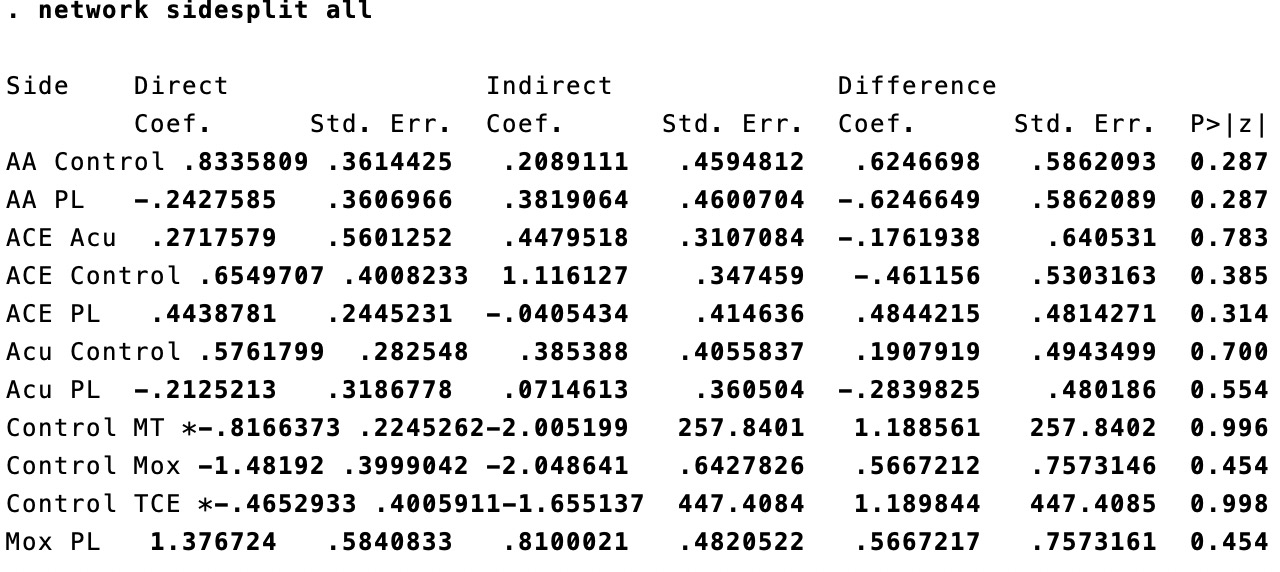


# Appendix 5 Result of network meta-analyses on non-pharmacological classes for WC

| **TCE**  (SUCRA= 33.2%) | - | - | - | - | - | - | - |
| --- | --- | --- | --- | --- | --- | --- | --- |
| 0.63 (-0.73,1.99) | **PL**  (SUCRA= 72.9%) | - | - | - | - | - | - |
| 1.01 (-0.18,2.20) | 0.38 (-0.97,1.73) | **Mox**  (SUCRA=  90.0%) | - | - | - | - | - |
| 0.35 (-0.62,1.33) | -0.28 (-1.45,0.88) | -0.66 (-1.62,0.30) | **MT**  (SUCRA= 56.7%) | - | - | - | - |
| -0.47 (-1.32,0.38) | **-1.10 (-2.16,-0.04)** | **-1.48 (-2.31,-0.64)** | **-0.82 (-1.30,-0.34)** | **Control**  (SUCRA= 3.6%) | - | - | - |
| 0.11 (-0.92,1.14) | -0.52 (-1.74,0.69) | -0.90 (-1.92,0.12) | -0.24 (-1.00,0.52) | 0.58 (-0.01,1.16) | **Acu**  (SUCRA= 39.2%) | - | - |
| 0.19 (-1.01,1.38) | -0.44 (-1.80,0.91) | -0.82 (-2.01,0.36) | -0.16 (-1.13,0.80) | 0.66 (-0.18,1.50) | 0.08 (-0.95,1.11) | **ACE**  (SUCRA= 47.1%) | - |
| 0.03 (-0.86,0.92) | **0.85 (0.10,1.60)** | 0.27 (-0.68,1.22) | 0.19 (-1.36,1.74) | -0.31 (-1.69,1.08) | 0.19 (-0.94,1.32) | -0.89 (-2.34,0.56) | **AA**  (SUCRA= 57.3%) |

Treatments are reported in order of efficacy post-treatment with ranking according to surface under the cumulative ranking curves. Comparisons between treatments should be read from left to right, and the estimate is in the cell in common between the column-defining intervention and the row-defining intervention. Efficacy post-treatment values are given as mean overall change in symptoms (mean differences [MDs]); MDs of more than 0 favor the row-defining intervention. Data in parentheses represent 95% credible intervals. To obtain MDs for comparisons in the opposite direction, negative values should be converted into positive values, and vice versa. Significant results are set in boldface. PL, placebo therapy. Control, usual care. TCE, traditional Chinese exercises. Mox, moxibustion. MT, manual therapy. ACE, acupoint catgut embedding. AA, auricular acupuncture. Acu, acupuncture.

# Appendix **6.1** Subgroup Analyses for BMI **and**WC

| dimension | BMI | | | WC | | |
| --- | --- | --- | --- | --- | --- | --- |
|  | N of studies | *I^2^* (%) | SMD(95%CI) | N of studies | *I^2^* (%) | SMD(95%CI) |
| Time | | | | | | |
| ≤4weeks | 8 | 0 | -0.50( -0.70, -0.30) | 7 | 59.8 | -0.80(-1.15, -0.45) |
| 4-8weeks | 19 | 81.2 | -0.68 (-0.93, -0.44) | 16 | 84.0 | -0.79(-1.07, -0.51) |
| ≥8weeks | 10 | 76.9 | -0.45( -0.74, -0.15) | 9 | 49.9 | -0.57(-0.77, -0.37) |
| N | | | | | | |
| ≤60 | 16 | 7.6 | -0.53(-0.69, -0.38) | 12 | 65.8 | -0.90 (-1.21, -0.60) |
| 60-100 | 14 | 83.5 | -0.74( -1.04, -0.43) | 12 | 56.5 | -0.70 (-0.90, -0.50) |
| ≥100 | 7 | 82.1 | -0.35( -0.65, -0.04) | 8 | 88.5 | -0.55 (-0.91, -0.19) |
| Frequency | | | | | | |
| ≤2 times a week | 19 | 70.9 | -0.50(-0.69, -0.32) | 18 | 71.9 | -0.66 (-0.86, -0.46) |
| 2-5 times a week | 12 | 84.4 | -0.70( -1.06, -0.34) | 10 | 81.8 | -0.86( -1.20, -0.51) |
| ≥5 times a week | 6 | 0 | -0.56( -0.79, -0.33) | 4 | 71.5 | -0.68( -1.18, -0.18) |

Appendix 6.2 **Regression analysis of BMI and WC**

| dimension | BMI | WC |
| --- | --- | --- |
|  | ß-coefficient (95%CrI) | ß-coefficient (95%CrI) |
| Time | -0.670(-4.479, 2.067) | -1.599(-4.362, 0.930) |
| N | -0.809(-2.290, 0.500) | -1.689(-3.899, 0.613) |
| Frequency | -0.530(-1.635,1.481) | 0.158(-2.293,3.010) |
| BMI baseline | -0.138(-1.187,0.789) | -0.386(-2.744,2.137) |

# **Appendix 7 A sensitivity network meta-analysis**

## **Appendix 7.1 A sensitivity network meta-analysis of BMI outcomes was performed by excluding studies with sample size ≤30 and intervention frequency ≥ 7 times/week**

| **TCE** | 0.39  (-0.24,1.03) | **-1.04**  **(-1.76,-0.33)** | -0.22  (-0.84,0.39) | 0.39  (-0.13,0.91) | 0.13  (-0.48,0.75) | -0.18  (-0.82,0.46) | -0.25  (-0.95,0.44) |
| --- | --- | --- | --- | --- | --- | --- | --- |
| -0.44  (-0.99,0.11) | **PL** | **-1.44**  **(-1.98,-0.90)** | **-0.62**  **(-1.10,-0.13)** | -0.01  (-0.37,0.35) | -0.26  (-0.57,0.05) | -0.57  (-0.87,-0.28) | -0.65  (-1.08,-0.21) |
| **1.01 (0.36,1.65)** | **1.45 (0.92,1.97)** | **Mox** | **0.82 (0.23,1.41)** | **1.43 (0.94,1.92)** | **1.18 (0.63,1.73)** | **0.86 (0.29,1.43)** | **0.79 (0.15,1.43)** |
| 0.19  (-0.35,0.72) | **0.63**  **(0.16,1.09)** | **-0.82**  **(-1.39,-0.25)** | **MT** | **0.61 (0.28,0.93)** | 0.36  (-0.10,0.82) | 0.04  (-0.45,0.53) | -0.03  (-0.59,0.53) |
| -0.42  (-0.85,0.01) | 0.02  (-0.32,0.36) | **-1.43**  **(-1.91,-0.95)** | **-0.61**  **(-0.92,-0.29)** | **Control** | -0.25  (-0.58,0.07) | **-0.57**  **(-0.94,-0.20)** | **-0.64**  **(-1.10,-0.19)** |
| -0.18  (-0.71,0.35) | 0.26  (-0.03,0.56) | **-1.18**  **(-1.72,-0.65)** | -0.36  (-0.81,0.08) | 0.25  (-0.07,0.56) | **Acu** | -0.31  (-0.66,0.03) | -0.39  (-0.87,0.09) |
| 0.14  (-0.42,0.69) | **0.58**  **(0.29,0.86)** | **-0.87**  **(-1.42,-0.32)** | -0.05  (-0.53,0.42) | **0.56 (0.20,0.91)** | 0.31  (-0.02,0.64) | **ACE** | -0.07  (-0.57,0.42) |
| 0.18  (-0.40,0.77) | **0.62**  **(0.22,1.03)** | **-0.83**  **(-1.42,-0.23)** | -0.01  (-0.51,0.50) | **0.60**  **(0.20,1.00)** | 0.36  (-0.09,0.80) | 0.04  (-0.41,0.50) | **AA** |

Treatment sample size ≤30 frequency ≥ 7 times/week

## **Appendix 7.2 A sensitivity network meta-analysis of WC outcomes was performed by excluding studies with sample size ≤30 and intervention frequency ≥ 7 times/week**

| **TCE** | 0.20  (-0.98,1.39) | -0.91  (-2.16,0.35) | -0.11  (-1.28,1.05) | 0.70  (-0.38,1.79) | 0.24  (-0.93,1.40) | -0.15  (-1.34,1.05) | 0.36  (-0.87,1.58) |
| --- | --- | --- | --- | --- | --- | --- | --- |
| 0.03  (-0.83,0.90) | **PL** | **-1.11**  **(-1.79,-0.42)** | -0.32  (-0.94,0.31) | **0.50 (0.03,0.97)** | 0.03  (-0.41,0.47) | -0.35  (-0.74,0.04) | 0.15  (-0.39,0.70) |
| **1.14 (0.19,2.10)** | **1.11 (0.44,1.78)** | **Mox** | **0.79 (0.04,1.55)** | **1.61 (0.98,2.23)** | **1.14 (0.43,1.85)** | **0.76 (0.03,1.49)** | **1.26 (0.46,2.06)** |
| 0.35  (-0.49,1.20) | 0.32  (-0.30,0.93) | **-0.79**  **(-1.53,-0.06)** | **MT** | **0.82 (0.40,1.24)** | 0.35  (-0.25,0.95) | -0.03  (-0.68,0.61) | 0.47  (-0.24,1.18) |
| -0.46  (-1.20,0.27) | **-0.50**  **(-0.96,-0.04)** | **-1.61**  **(-2.22,-1.00)** | **-0.81**  **(-1.23,-0.40)** | **Control** | **-0.47**  **(-0.89,-0.04)** | **-0.85**  **(-1.34,-0.36)** | -0.35  (-0.92,0.22) |
| 0.00  (-0.84,0.85) | -0.03  (-0.46,0.40) | **-1.14**  **(-1.84,-0.44)** | -0.35  (-0.93,0.24) | **0.47 (0.05,0.88)** | **Acu** | -0.38  (-0.87,0.11) | 0.12  (-0.50,0.75) |
| 0.38  (-0.50,1.26) | 0.35  (-0.03,0.73) | **-0.76**  **(-1.48,-0.05)** | 0.03  (-0.60,0.66) | **0.85 (0.37,1.33)** | 0.38  (-0.10,0.86) | **ACE** | 0.50  (-0.12,1.13) |
| -0.12  (-1.04,0.81) | -0.15  (-0.69,0.39) | **-1.26**  **(-2.04,-0.48)** | -0.47  (-1.16,0.23) | 0.35 (-0.21,0.91) | -0.12  (-0.73,0.49) | -0.50  (-1.11,0.12) | **AA** |

Treatment sample size ≤30 frequency ≥ 7 times/week

BMI

**WC**

**BMI**

#
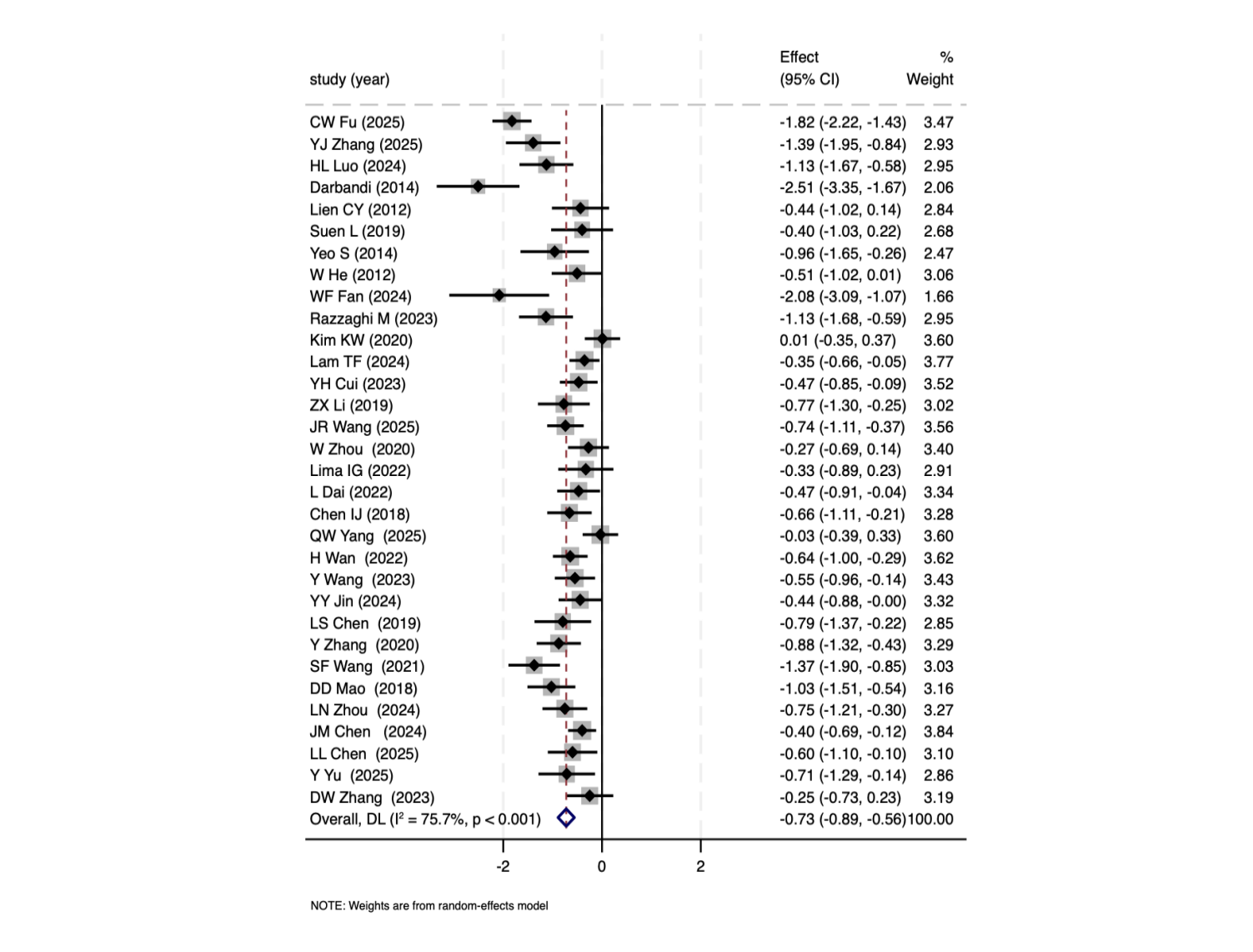
 Appendix 8 BMI、WC Forest Plot


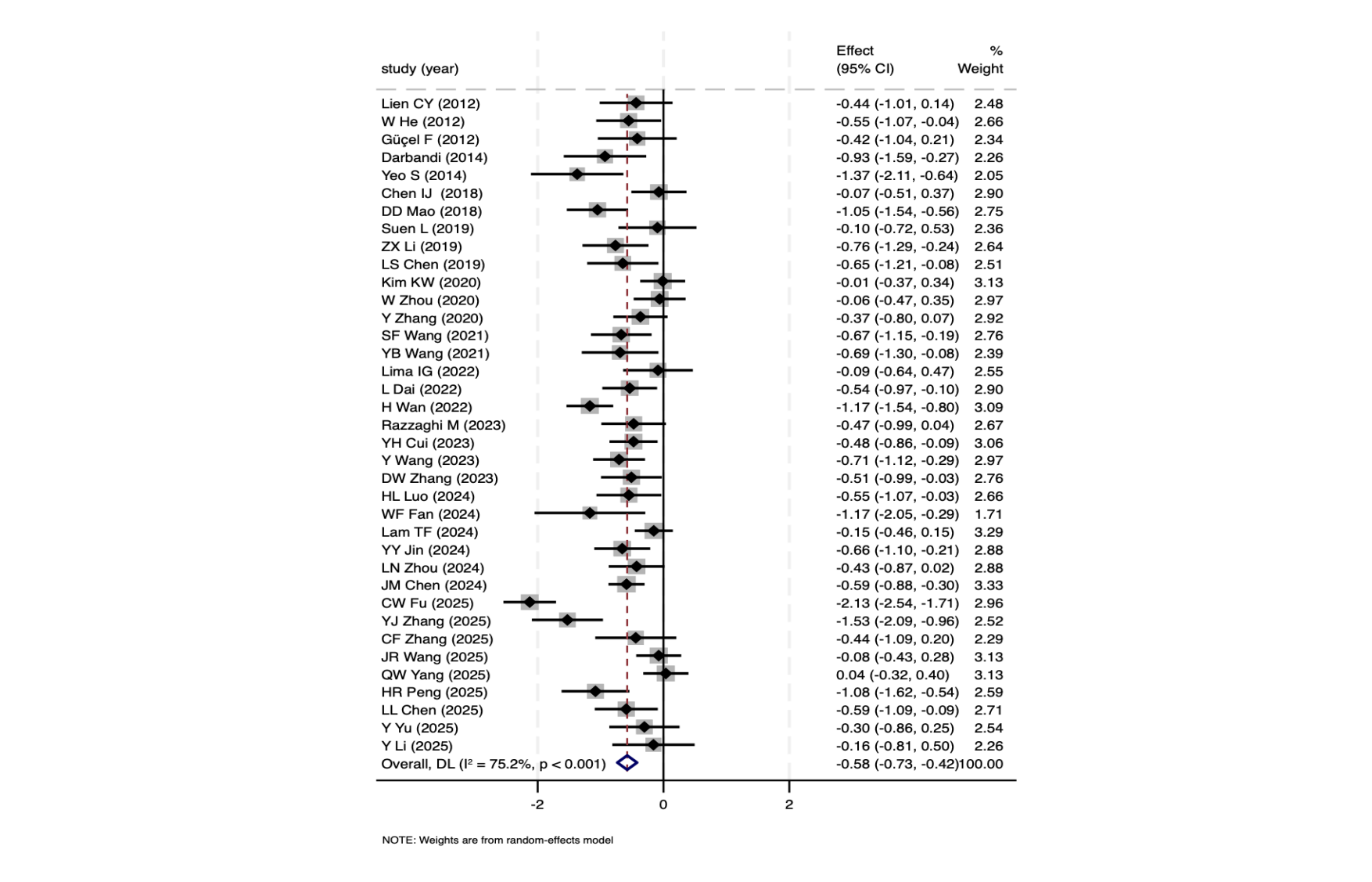


BMI

WC

# Appendix 9 Grade of evidence assessment using the CINeMA (Confidence in Network Meta

## Appendix 9-1 CINEMA Assessments for BMI

| **Comparison** | **N of**  **studies** | **Within-**  **study bias** | **Reporting**  **bias** | **Indirectness** | **Imprecision** | **Heterogeneity** | **Incoherence** | **Confidence**  **rating** |
| --- | --- | --- | --- | --- | --- | --- | --- | --- |
| AA vs. Control | 4 | Some concerns | Low risk | No concerns | No concerns | Some concerns | No concerns | Low |
| AA vs. PL | 3 | Some concerns | Low risk | Some concerns | No concerns | Some concerns | No concerns | Very low |
| ACE vs.ACU | 2 | Some concerns | Low risk | Some concerns | No concerns | Some concerns | No concerns | Very low |
| ACE vs. Control | 2 | Some concerns | Low risk | No concerns | No concerns | Some concerns | No concerns | Low |
| ACE vs. PL | 5 | Some concerns | Low risk | Some concerns | No concerns | Some concerns | No concerns | Very low |
| ACU vs. Control | 4 | Major concerns | Low risk | No concerns | No concerns | Some concerns | No concerns | Very low |
| ACU vs. PL | 4 | Some concerns | Low risk | Some concerns | No concerns | Some concerns | No concerns | Very low |
| Control vs. MT | 6 | Major concerns | Low risk | No concerns | No concerns | Some concerns | Major concerns | Very low |
| Control vs. Mox | 2 | Some concerns | Low risk | No concerns | No concerns | No concerns | No concerns | Moderate |
| Control vs. TCE | 4 | Major concerns | Low risk | No concerns | Some concerns | No concerns | Major concerns | Very low |
| Mox vs. PL | 1 | Some concerns | Low risk | Some concerns | No concerns | No concerns | No concerns | Low |
| AA vs. ACE | 0 | Some concerns | Low risk | Some concerns | No concerns | Some concerns | Major concerns | Very low |
| AA vs. ACU | 0 | Some concerns | Low risk | No concerns | Some concerns | No concerns | Major concerns | Very low |
| AA vs. MT | 0 | Major concerns | Low risk | No concerns | No concerns | Some concerns | Major concerns | Very low |
| AA vs. Mox | 0 | Some concerns | Some concerns | No concerns | No concerns | Some concerns | Major concerns | Very low |
| AA vs. TCE | 0 | Some concerns | Low risk | No concerns | Some concerns | No concerns | Major concerns | Very low |
| ACE vs. MT | 0 | Major concerns | Low risk | No concerns | No concerns | Some concerns | Major concerns | Very low |
| ACE vs. Mox | 0 | Some concerns | Low risk | No concerns | No concerns | Some concerns | Major concerns | Very low |
| ACE vs. TCE | 0 | Some concerns | Some concerns | No concerns | No concerns | Some concerns | Major concerns | Very low |
| ACU vs. MT | 0 | Major concerns | Some concerns | No concerns | Some concerns | No concerns | Major concerns | Very low |
| ACU vs. Mox | 0 | Some concerns | Low risk | No concerns | No concerns | No concerns | Major concerns | Very low |
| ACU vs. TCE | 0 | Major concerns | Low risk | No concerns | No concerns | Some concerns | Major concerns | Very low |
| Control vs. PL | 0 | Some concerns | Low risk | Some concerns | No concerns | No concerns | Major concerns | Very low |
| MT vs. Mox | 0 | Major concerns | Low risk | No concerns | No concerns | Some concerns | Major concerns | Very low |
| MT vs. PL | 0 | Some concerns | Low risk | No concerns | No concerns | Some concerns | Major concerns | Very low |
| MT vs. TCE | 0 | Major concerns | Low risk | No concerns | No concerns | Some concerns | Major concerns | Very low |
| Mox vs. TCE | 0 | Some concerns | Low risk | No concerns | No concerns | No concerns | Major concerns | Very low |
| PL vs. TCE | 0 | Some concerns | Some concerns | No concerns | Some concerns | No concerns | Major concerns | Very low |

PL, placebo therapy. Control, usual care. TCE, traditional Chinese exercises. Mox, moxibustion. MT, manual therapy. ACE, acupoint catgut embedding. AA, auricular acupuncture. Acu, acupuncture.

## Appendix 9-2 CINEMA Assessments for WC

| **Comparison** | **N of**  **studies** | **Within-**  **study bias** | **Reporting**  **bias** | **Indirectness** | **Imprecision** | **Heterogeneity** | **Incoherence** | **Confidence**  **rating** |
| --- | --- | --- | --- | --- | --- | --- | --- | --- |
| AA vs. Control | 3 | Some concerns | Low risk | No concerns | No concerns | No concerns | No concerns | Moderate |
| AA vs. PL | 3 | Some concerns | Low risk | Some concerns | No concerns | No concerns | No concerns | Low |
| ACE vs. ACU | 1 | Some concerns | Low risk | Some concerns | No concerns | Some concerns | No concerns | Very low |
| ACE vs. Control | 2 | Some concerns | Low risk | No concerns | No concerns | Some concerns | No concerns | Low |
| ACE vs. PL | 5 | Some concerns | Low risk | Some concerns | No concerns | Some concerns | No concerns | Very low |
| ACU vs. Control | 4 | Major concerns | Low risk | No concerns | No concerns | Some concerns | No concerns | Very low |
| ACU vs. PL | 3 | Some concerns | Low risk | Some concerns | No concerns | Some concerns | No concerns | Very low |
| Control vs. MT | 6 | Major concerns | Low risk | No concerns | No concerns | No concerns | Major concerns | Very low |
| Control vs. Mox | 2 | Some concerns | Low risk | No concerns | No concerns | No concerns | No concerns | Moderate |
| Control vs. TCE | 2 | Major concerns | Low risk | No concerns | Some concerns | No concerns | Major concerns | Very low |
| Mox vs. PL | 1 | Some concerns | Low risk | Some concerns | No concerns | No concerns | No concerns | Low |
| AA vs. ACE | 0 | Some concerns | Low risk | Some concerns | No concerns | Some concerns | Major concerns | Very low |
| AA vs. ACU | 0 | Some concerns | Low risk | No concerns | Some concerns | No concerns | Major concerns | Very low |
| AA vs. MT | 0 | Major concerns | Low risk | No concerns | No concerns | Some concerns | Major concerns | Very low |
| AA vs. Mox | 0 | Some concerns | Low risk | No concerns | Some concerns | No concerns | Major concerns | Very low |
| AA vs. TCE | 0 | Some concerns | Some concerns | No concerns | Some concerns | No concerns | Major concerns | Very low |
| ACE vs. MT | 0 | Major concerns | Low risk | No concerns | No concerns | Some concerns | Major concerns | Very low |
| ACE vs. Mox | 0 | Some concerns | Low risk | No concerns | No concerns | No concerns | Major concerns | Very low |
| ACE vs. TCE | 0 | Some concerns | Low risk | No concerns | No concerns | Major concerns | Major concerns | Very low |
| ACU vs. MT | 0 | Major concerns | Some concerns | No concerns | No concerns | Some concerns | Major concerns | Very low |
| ACU vs. Mox | 0 | Some concerns | Low risk | No concerns | No concerns | No concerns | Major concerns | Very low |
| ACU vs. TCE | 0 | Major concerns | Some concerns | No concerns | No concerns | Major concerns | Major concerns | Very low |
| Control vs. PL | 0 | Some concerns | Low risk | No concerns | No concerns | No concerns | Major concerns | Very low |
| MT vs. Mox | 0 | Major concerns | Low risk | No concerns | No concerns | Some concerns | Major concerns | Very low |
| MT vs. PL | 0 | Some concerns | Low risk | No concerns | No concerns | Some concerns | Major concerns | Very low |
| MT vs. TCE | 0 | Major concerns | Low risk | No concerns | Some concerns | No concerns | Major concerns | Very low |
| Mox vs. TCE | 0 | Some concerns | Low risk | No concerns | No concerns | No concerns | Major concerns | Very low |
| PL vs. TCE | 0 | Some concerns | Low risk | No concerns | Some concerns | No concerns | Major concerns | Very low |
